# Stromal cells regulate mechanics of tumour spheroid

**DOI:** 10.1016/j.mtbio.2023.100821

**Published:** 2023-10-06

**Authors:** Ayushi Agrawal, Soufian Lasli, Yousef Javanmardi, Diane Coursier, Auxtine Micalet, Sara Watson, Somayeh Shahreza, Bianca Serwinski, Boris Djordjevic, Nicolas Szita, Umber Cheema, Sergio Bertazzo, Fernando Calvo, Emad Moeendarbary

**Affiliations:** a Department of Mechanical Engineering, University College London, London, WC1E 7JE, UK; b Instituto de Biomedicina y Biotecnología de Cantabria (Consejo Superior de Investigaciones Científicas, Universidad de Cantabria), Santander, Spain; c Department of Targeted Intervention, Division of Surgery and Interventional Science, University College London, London WC1E 7JE, UK; d 199 Biotechnologies Ltd, Gloucester Road, London, W2 6LD, UK; e Faculty of Social Sciences, Northeastern University London, London, E1W 1LP, UK; f Department of Biochemical Engineering, University College London, London WC1E 7JE, UK; g Department of Medical Physics and Biomedical Engineering, University College London, London, WC1E 6BT, UK; h Department of Biological Engineering, Massachusetts Institute of Technology, Cambridge, Massachusetts 02139, USA

**Keywords:** Traction forces, Contractility, Stromal cells, Cancer spheroids, Mechanobiology

## Abstract

The remarkable contractility and force generation ability exhibited by cancer cells empower them to overcome the resistance and steric hindrance presented by a three-dimensional, interconnected matrix. Cancer cells disseminate by actively remodelling and deforming their extracellular matrix (ECM). The process of tumour growth and its ECM remodelling have been extensively studied, but the effect of the cellular tumour microenvironment (TME) has been ignored in most studies that investigated tumour-cell-mediated ECM deformations and realignment. This study reports the integration of stromal cells in spheroid contractility assays that impacts the ECM remodelling and invasion abilities of cancer spheroids. To investigate this, we developed a novel multilayer *in vitro* assay that incorporates stromal cells and quantifies the contractile deformations that tumour spheroids exert on the ECM. We observed a negative correlation between the spheroid invasion potential and the levels of collagen deformation. The presence of stromal cells significantly increased cancer cell invasiveness and altered the cancer cells' ability to deform and realign collagen gel, due to upregulation of proinflammatory cytokines. Interestingly, this was observed consistently in both metastatic and non-metastatic cancer cells. Our findings contribute to a better understanding of the vital role played by the cellular TME in regulating the invasive outgrowth of cancer cells and underscore the potential of utilising matrix deformation measurements as a biophysical marker for evaluating invasiveness and informing targeted therapeutic opportunities.

## Graphical abstract

**Figure:**
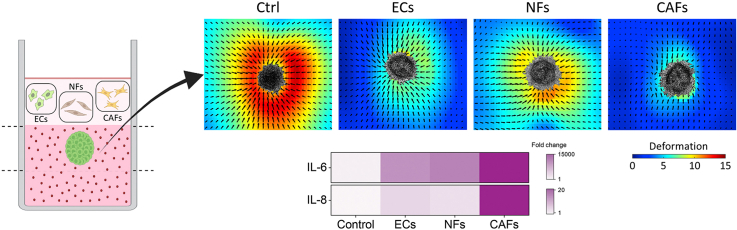

## 1 Introduction

Tumour growth and metastasis are inherently mechanical processes that involve physical interactions and the movement of malignant cells from their primary tumour site to distant tissues. Before the onset of metastasis, cancer cells interact with their tumour microenvironment (10.13039/501100010577TME), including the extracellular matrix (ECM) and neighbouring stromal cells, to support their growth and local invasion [1]. In this process, the cells generate forces and remodel the ECM through cell-matrix interactions, which enables cancer cells to navigate through the dynamic TME and propagate throughout the body [2]. Understanding these interactions in detail has the potential to curb the spread of cancer cells at the primary site, thereby facilitating early intervention. There is therefore a growing need to develop advanced *in vitro* models that mimic the tumour and its microenvironment to study cellular processes in physiologically relevant settings, opening the way for better therapeutic drug targeting.

The spatial complexity of the tumour *in vivo* makes the capture and study of cell-generated tractions on the ECM challenging. Several *in vitro* assays measuring the traction forces generated by single cancer cells, or collectively as multicellular cancer spheroids, have emerged. Traction force microscopy on single cells has been useful in correlating the metastatic potential of cancer cells with their cellular traction stresses [[3], [4], [5]]. However, these assays frequently utilise synthetic polymers, such as polyacrylamide, or silicone-based elastomers like PDMS, as linearly elastic substrate in 2D to measure traction stresses. Subsequent studies have made considerable improvements through the use of collagen, a natural hydrogel and a major component of the ECM, for quantifying deformations in spheroids [6]. Studies of collagen embedded with multicellular head and neck squamous cell carcinoma spheroids have observed a direct correlation between the spheroid's invasive ability and the mechanical remodelling process of the ECM [7]. Another such model showed the active radial realignment of the collagen fibres through tension generation in the fibres by the contractile forces originating from cancer spheroids [8]. However, these *in vitro* models do not incorporate the cellular components of the TME (such as the stromal cells) that can also modulate the invasiveness and deformation fields of the cancer spheroids.

These stromal cells form a heterogeneous population of non-cancerous cells in the tumour vicinity. These include, for example, endothelial cells, fibroblasts, cancer-associated fibroblasts (CAFs), pericytes, mesenchymal stem cells, and immune cells [[9], [10], [11]]. These cells modulate the metastatic transition of cancer cells through dynamic biochemical and biophysical feedback signals [12]. Biochemical signals include the secretion of growth factors (VEGF, EGF, FGF, PDGF), pro-inflammatory cytokines (IL-6, IL-8, TNF) [13] and pro-metastatic chemokines (CXCL8, CCL2, CCL5) [14], among others, that induce tumour proliferation and motility. Biophysical factors include the topographic reconfiguration of the stroma, such as ECM fibre alignment, increased stiffness, elevated solid stress, and the compression of blood vessels that fuel cancer progression [2]. CAFs in particular have gained much attention as a key component of the TME that promote tumour invasion and metastasis through both physical and chemical crosstalk with the tumour cells [[15], [16], [17]]. However, to our knowledge, little information is available on how the biochemical interactions between stromal and cancer cells affect the ECM remodelling ability of the cancer cells. Thus, characterising these interactions is important to advance our understanding of the potential interplay of chemical cues between a tumour and its stroma that influence the tumour's physical capacity for collagen deformation and invasion.

In this study, we investigated the effect of stromal cells on a cancer spheroid's ability to realign collagen gel and, thereafter, invade. We used a multilayer *in vitro* assay employing traction force microscopy to quantify the displacements generated by the cancer spheroids and correlate these displacements with the levels of invasiveness of the cancer spheroids into the surrounding collagen ECM. Using this assay, we then explored the modulatory effect of stromal cells such as endothelial cells (ECs), normal fibroblasts (NFs), and CAFs, critical and abundant components of the cellular TME. In addition, we also compared metastatic (SK-MES-1) and non-metastatic (A549) lung cancer cell lines. Finally, molecular characterisation of the system identified several growth factors and cytokines that were dysregulated as a result of the stromal-tumour cell interaction that enhanced the invasion capability of the cancer spheroids. To the best of our knowledge, this work is the first to integrate biochemical signals from stromal cells with the deformations generated by the cancer cells that remodel the ECM in a traction microscopy assay.

## 2 Material and methods

### 2.1 Cell culture

GFP-A549 (Sigma) cells were cultured in F-12k medium (Thermo Fisher Scientific) supplemented with 10% FBS and 1% penicillin-streptomycin solution and used for no more than 15 passages. SK-MES-1 (Cell Lines Service, CLS) cells were cultured in ATCC-formulated Eagle's Minimum Essential Medium (ATCC), supplemented with 10% FBS and 1% penicillin-streptomycin solution and used for no more than 10 passages. Human Umbilical Vein ECs (Lonza) were cultured in Endothelial Growth Medium (EGM-2MV, Lonza), and passages 6 to 8 were used for the experiments. Normal Human Lung Fibroblasts (NFs, Lonza) were cultured in Fibroblast Growth Medium (FGM-2, Lonza) and used up to passage 8 for all the experiments. Human Lung CAFs were cultured in DMEM, supplemented with 10% FBS and 1% penicillin-streptomycin solution and used up to 15 passages. All cells were maintained at 37 ᵒC and 5% CO_2_ in humid incubators.

### 2.2 Spheroid formation

A549 and SK-MES-1 cells were disassociated from the cell culture dish using trypsin-EDTA (0.05%, HyClone), spun-down, and counted (10,000 cells/ml). The cells were resuspended in their respective cell culture growth medium as mentioned before, supplemented with 20% Methocel solution [18] and seeded to approximately 1000 cells/well in a 96-well U-bottom plate. The plate was then centrifuged at 350 rcf for 10 min and subsequently maintained in a humidified incubator at 37 ᵒC, 5% CO_2_. The spheroids were ready to embed into the collagen gel after 24 h.

### 2.3 Collagen and fibrin hydrogel preparation

Collagen hydrogel was prepared as an unpolymerised solution on ice under sterile conditions, using pre-cooled sterile water, pre-washed 4 μm fluorescent beads (FluoSpheres Sulfate Microspheres, F8858, Thermo Fisher Scientific), HEPES buffer, 10× PBS-phenol red, NaOH (0.5 M) and rat-tail Collagen I (VWR International). These solutions were mixed such that the working collagen I concentration was 2 mg/ml at pH 7.4. The mixture was always prepared fresh before setting up an experiment.

Fibrin hydrogel was prepared by mixing equal volumes of thrombin and fibrinogen solution. Fibrinogen working solution was prepared by reconstituting 6 mg/ml bovine fibrinogen powder (Sigma) in PBS without calcium and magnesium in a water bath at 37 ᵒC for approximately 2 h. The solution was then filter-sterilised (through a 2 μm filter) and stored at 4 ᵒC for no more than one week. Thrombin stock solution was prepared by dissolving thrombin (1 KU, Sigma) in 10 ml PBS without calcium and magnesium. This solution was filter-sterilised and labelled aliquots were stored at −20 ᵒC for up to 6 months. The thrombin working solution (4 U/ml) was prepared just before setting up the experiment in EGM-2MV media over ice.

### 2.4 Spheroid contractility assay setup

**Fig. 1:**
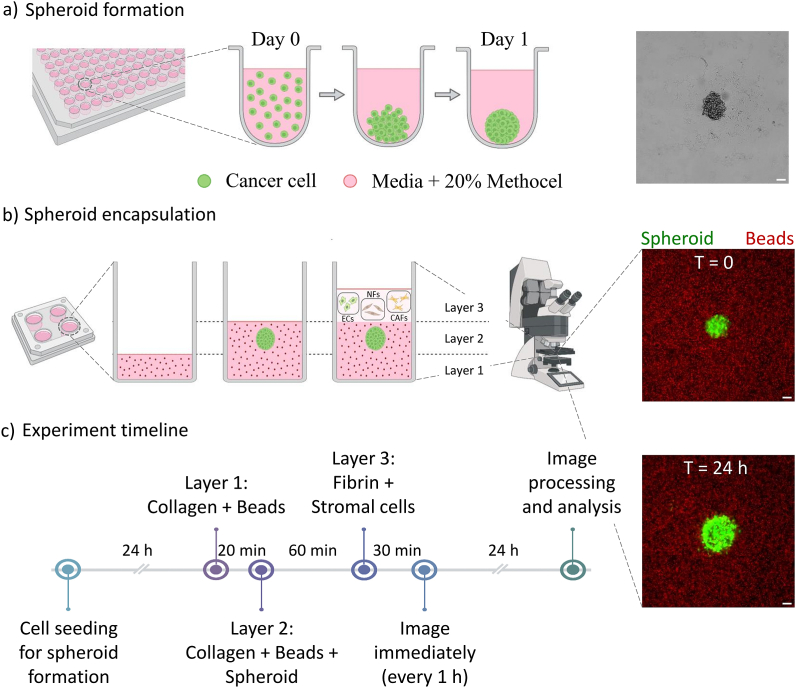
**Multilayer spheroid contractility assay.** (a) Schematic representation of spheroid formation in a 96-well U-bottom well plate. The image on the right shows a spheroid formed at Day 1 post cell seeding. (b) Schematic representation of the process of spheroid encapsulation. Different layers can be distinguished: Layer 1 (collagen and beads), Layer 2 (collagen, beads, and spheroid), Layer 3 (fibrin and stromal cells). Stromal cells considered in the study are - ECs: Human Umbilical Vein Endothelial Cells, NFs: Normal Human Lung Fibroblasts, CAFs: Human Lung Cancer Associated Fibroblasts. The image on the right is a representative image of an A549 spheroid embedded with beads in collagen hydrogel at t = 0 h. (c) Experimental timeline. The image on the right is a representative image of an A549 spheroid embedded with beads in collagen hydrogel at t = 24 h. Scale bar is 100 μm.

The schematic in Fig. 1 was constructed using BioRender (biorender.com). Fig. 1 b shows the spheroid encapsulation process in collagen gel, followed by stromal cell embedding. The hydrogel solutions were kept on ice during the entire cell seeding process. Layer 1 in the 4-well dish (Nunc, 10507591, Fisher Scientific) was prepared by dispensing approximately 200 μl/well of unpolymerised collagen hydrogel solution (containing the fluorescent beads), followed by incubation in a humidity box at 37 ᵒC. This layer was formed to prevent the spheroids from adhering to the plastic bottom of the dish. After 30 min, a mixture of maximum 3 spheroids and unpolymerised collagen solution (200 μl/well, containing the fluorescent beads) was poured over layer 1, manually locating the spheroids near the centre of the well, while also making sure that the spheroids were well separated (>3 mm). Samples with distance smaller than this were discarded. This mixture was then incubated for 1 h in the incubator. Meanwhile, the stromal cells (ECs, NFs, or CAFs) were disassociated and counted separately (0.5 × 10^6^ cells/ml), centrifuged, and resuspended in thrombin working solution. An equal volume of the thrombin-cell suspension was mixed with fibrinogen working solution to achieve a final concentration of 0.25 × 10^6^ cells/ml. Approximately 300 μl/well of this mixture was immediately poured over layer 2. The seeded dishes were again incubated for 30 min, before filling the dish with pre-warmed EGM2-MV medium (600 μl/well) for all conditions.

### 2.5 Fixation and collagen staining

To perform staining, the medium was aspirated from the dishes and the wells were washed (×2) with PBS. The wells were fixed with 4% PFA (Thermo Fisher Scientific) for 15 min and washed with PBS (x3, 5 min each). The collagen fibres were stained with 50 μM 5(6)-carboxytetramethylrhodamine succinimidyl ester (TAMRA-SE, Invitrogen) at 4 ᵒC overnight and rinsed 5 times with PBS.

### 2.6 Imaging

For particle image velocimetry (PIV) analysis, the dishes were imaged soon after cell seeding using an inverted epifluorescent microscope (Leica DMi8) equipped with a motorised stage and live-cell imaging modules (including a temperature and CO_2_ controller). High-resolution images (1392 × 1040 pixels) were recorded using a 5x objective with 150 μm z-stacks (15 μm separation between slices) acquired every 1 h per spheroid. For endpoint collagen fibre imaging, the images were captured using an upright confocal microscope (Zeiss 980 with Airyscan-2), with a 60x objective, after carefully removing the stromal cell layer using a scalpel to visualise the spheroids in Layer 2.

### 2.7 PIV and cell invasion image quantification

Collagen deformation was measured through fluorescent bead displacements and was quantified using a PIV plugin in ImageJ (Fiji). The dimension of the fluorescent beads (4 μm) was comparable to the pore size of a 2 mg/ml collagen matrix (∼1–2 μm) [19], which significantly decreased the chances of local diffusion arising from floating beads. Hence, displacement of the beads was largely caused by the collective collagen deformation generated by the individual spheroids. After image acquisition, all time points except t = 1 h were considered and all the image z-stacks were processed through the PIV plugin and saved as .txt files. Post processing of the data was performed using MATLAB (MathWorks). The data were drift-corrected, before the successive addition of displacements for the cumulative displacement calculation. A maximum displacement projection was then obtained as an average over all the slices. The average radial displacement in Fig. 3 a,c was calculated as the average of the displacements within approximately 150 μm of the spheroid surface. In addition, the normalised radial displacements were calculated by dividing the displacements relative to the spheroid major axis.

To quantify cell invasion, bright-field images of the spheroids were processed in ImageJ. The area of the spheroid spread was calculated by manually encircling the spheroid periphery (using the “freehand selection” tool in ImageJ) at different time points. The invasion index was calculated as the area of the spheroid at a given time divided by its area at t = 1 h.

### 2.8 Collagen angular distribution and fibre alignment measurements

To calculate the collagen angular distribution, the angle between the collagen fibres and the nearest spheroid periphery was considered. We used CurveAlign software, an open-source tool that employs a curvelet-based algorithm to measure the fibre orientation and degree of alignment. The methodology to measure collagen fibre distribution has been mentioned in detail elsewhere [20,21]. Briefly, the inputs for this software were a TAMRA-stained collagen fibre image and a spheroid boundary mask. To generate a spheroid boundary mask, the “freehand selection” tool in ImageJ was used to draw the boundary of the spheroid from the TAMRA-stained collagen fibre, and the “clear outside” function removed the unwanted background before a "threshold" was set. The image was converted to a mask and saved as a.tiff file. In the CurveAlign software, the collagen fibres were detected within a region of 100 μm from the spheroid boundary and the angles were retrieved from the Excel file saved by the software.

The collagen fibre alignment was measured by calculating the kurtosis coefficient for the angular distribution data. The kurtosis values were also obtained from the CurveAlign software.

### 2.9 Multiplex assay

For the sample preparation of the multiplex assay, after the experiment, the medium of each well was collected and spun at 3000 ×*g* prior to aliquoting to remove debris. The supernatant was frozen at −80 ᵒC and used for further testing. The multiplex assay performed was a Human Cytokine/Chemokine Panel A 48-Plex Discovery Assay (HD48A) conducted at Eve Technologies (Calgary, Alberta, Canada). A heatmap was generated using OriginPro. The data were represented as a fold change, i.e., the concentration of the cytokine in the stromal cell case was divided by the corresponding cytokine concentration in the control.

### 2.10 Quantification and statistical analysis

At least seven samples from three independent experimental repeats were analysed for quantification and statistical comparison. Each sample represents average of spheroids per well. Data were presented as mean ± s.e.m. Student's t-test was employed to evaluate statistical significance and p-values < 0.05 were considered statistically significant (*p < 0.05, **p < 0.01, and ***p < 0.001). Data analysis was performed using MATLAB (MathWorks) or OriginPro (OriginLab).

## 3 Results

### 3.1 Development of a multi-layer spheroid contractility assay

To study the effect of stromal cells on biophysical parameters associated with cancer invasion, we modified a previously described spheroid contractility assay [6] to incorporate the stromal cell compartment. Briefly, multicellular spheroids were formed by suspending cancer cells in a U-shaped 96-well plate (Fig. 1 a). The spheroids, similar in size and aspect (Supplementary Fig. 1), were mixed with 4 μm fluorescent fiducial marker beads in the unpolymerised collagen solution and poured onto a pre-coated collagen layer (also called layer 1) in a 24-well plate to polymerise. It was ensured that the spheroids were located near the centre of the well and the distance amongst each was relatively high (>3 mm) to avoid any edge-effects and spheroid-spheroid interactions, respectively, thus reducing variability in data collection. The layer 1 prevents the spheroids from attaching to the plastic bottom of the plate. A third layer (layer 3) of stromal cells (no cells (Control), ECs, NFs, or CAFs) were encapsulated in fibrin hydrogel and introduced on top of the spheroid-laden collagen hydrogel layer. The stromal cell concentrations were optimised such that they do not penetrate into layer 2 and interfere with bead tracking measurements. We observed no significant difference in stromal cell number within the experimental timeline (Supplementary Fig. 2). Fibrin gel was chosen for layer 3 as it reduces fibroblast contractility, thereby avoiding excessive gel shrinkage. This is in contrast to collagen, which tends to shrink substantially when embedded with fibroblasts [22], making it difficult to perform bead tracking analysis. Besides, we observed no significant differences in the fibre diameter (Supplementary Fig. 3), stiffness [23,24] (Supplementary Fig. 4), and diffusion transport properties [25] (Supplementary Fig. 5) between acellular collagen and fibrin hydrogels. After polymerisation, the hydrogel layers in the multilayer assay were well-integrated to each other, with the corresponding hydrogel heights - layer 1 (870.4 ± 32.4 μm), layer 2 (941.1 ± 69.8 μm), layer 3 (1451.8 ± 218.7 μm), irrespective of experimental conditions (Supplementary Fig. 6). The spheroids were imaged immediately after the gel polymerisation every 1 h for 24 h using an inverted epifluorescent microscope. The spheroid-induced deformations were tracked via the fluorescent bead displacements using Fiji. The displacement fields were reported in this study instead of the forces generated by spheroids because of the complexity to model mechanical properties (viscoelastic and viscoplastic nature) of the hydrogel accurately [26,27]. In addition, we assumed a uniformly arranged 3D space of collagen fibres around the cancer spheroids, as the collagen pore size is significantly smaller than the spheroid itself. The reproducibility of the results was ensured using sufficient experimental replicates.

### 3.2 Stromal cells reduce spheroid-generated ECM deformations

**Fig. 2:**
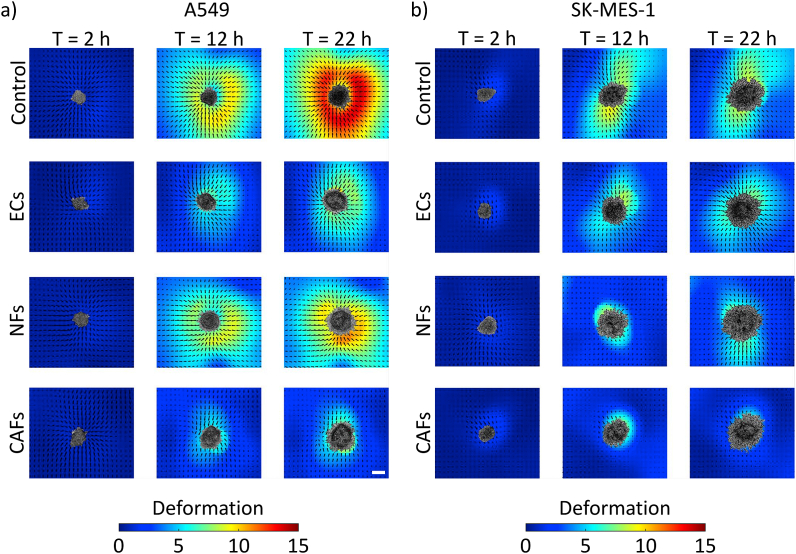
**Quantitative 2D mapping of cumulative collagen deformations.** Maps of the deformation field obtained by particle image velocimetry at 2 h, 12 h, and 22 h, after setting up the assay. Each row represents the effect of different stromal cells on the spheroid's ECM contracting ability in (a) non-metastatic (A549) and (b) metastatic (SK-MES-1) lung cancer cells. The arrow heads indicate the displacement vectors and the colour scale represents the displacement magnitude. Scale = 100 μm. (For interpretation of the references to color in this figure legend, the reader is referred to the Web version of this article.)

To quantify ECM deformations, we compared the spheroids derived from a metastatic (SK-MES-1) and a non-metastatic (A549) lung cancer cell line. Fig. 2 shows representative images of the quantitative time-dependent plots of cumulative collagen deformations in 2D (x,y) generated by the cancer spheroids. In case of controls, the cumulative collagen deformations observed in non-metastatic spheroids were higher compared to metastatic spheroids in terms of the magnitude of cumulative displacement. The raw radial deformations have been mentioned in Supplementary Figs. 7 and 8. In all the cases, collagen displacements were observed in the radial direction, suggesting a pulling effect on the collagen fibres. The ECM contraction deformation was observed shortly after the spheroids were embedded in the collagen gel and the deformations gradually reduced over time (Supplementary Figs. 7 and 8), as also seen previously [7].

**Fig. 3:**
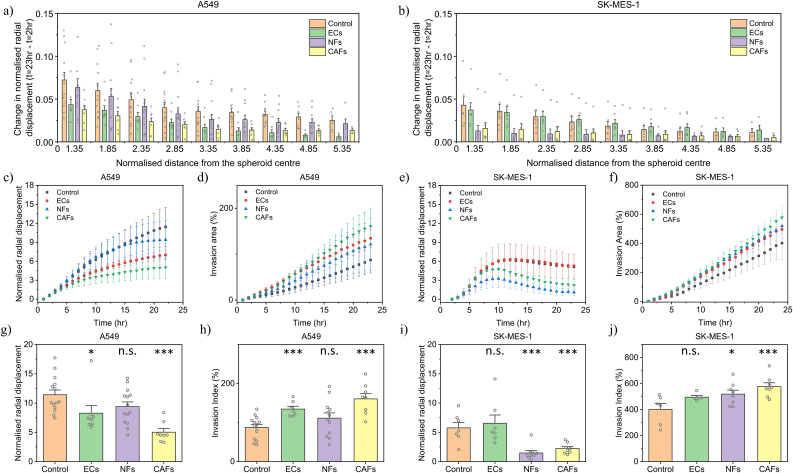
**Quantitative plots comparing collagen deformation and invasion of metastatic (SK-MES-1) and non-metastatic (A549) spheroids.** (a&b) Change in normalised radial displacement between t = 23 h and t = 2 h over the normalised distance from the spheroid centre for different cases of stromal cells in (a) A549 spheroids and (b) SK-MES-1 spheroids. (c&e) Average normalised radial displacement vs time for different cases of stromal cells in (c) A549 spheroids and (e) SK-MES-1 spheroid. (d&f) Invasion area (%) vs time for different stromal cell conditions in (d) A549 spheroids and (f) SK-MES-1 spheroids. (g&i) Average normalised displacement at t = 23 h in (g) A549 spheroids and (i) SK-MES-1 spheroids. (h&j) Invasion area (%) at t = 23 h in (h) A549 spheroids and (j) SK-MES-1 spheroids. Each data point represents average of spheroids per well. The plots and error bars in (c)–(f) represent mean ± s.d. and from (g)–(j) represent mean ± s.e.m. for A549 (n = 14 for Ctrl, n = 8 for ECs, n = 12 for NFs, n = 8 for CAFs) and SK-MES-1 (n = 7 for Ctrl, n = 7 for ECs, n = 9 for NFs, n = 8 for CAFs) spheroids and *p < 0.05, **p < 0.01, and ***p < 0.001 (Student's t-test).

The case with no stromal cells in layer 3 was established as a control and compared with stromal cells, ECs, NFs, and CAFs. We observed that the presence of stromal cells altered the collagen deformation field generated by the spheroids. In both the A549 and SK-MES-1 cases, the control groups showed maximum deformation fields whereas the CAFs group showed minimum displacements (Fig. 2).

### 3.3 Collagen deformation correlates with spheroid invasion capability

To study the dynamic rearrangement of the ECM, we quantified the collagen deformation over time in terms of cumulative radial displacements (i.e., the sum of the magnitudes of consecutive displacement vectors) for all cases in both metastatic and non-metastatic cell lines. These displacements were maximum near the tumour periphery (0.072 ± 0.008 for A549, 0.015 ± 0.006 for SK-MES-1) and gradually decreased (0.025 ± 0.002 for A549, 0.023 ± 0.004 for SK-MES-1) with increasing distance from the spheroid centre (Fig. 3 a,b, Supplementary Fig. 9 a,b). This suggests that spheroids could be the source of the observed deformations. We observed that the onset of emerging differences in the displacement profile of different stromal cell cases, in both cancer cell lines, occurred after t = 5–7 h post-imaging, coinciding with the time that stromal cells take to adhere and adapt to the fibrin gel and release potential soluble modulators during cell-cell interactions.

Despite the proliferation of the cells within the tumour spheroids, leading to growth and compression of the surrounding collagen, this effect (through outward-directed deformations) was not observed in any of the cases of the A549 spheroids. Fig. 3 c shows monotonically increasing cumulative radial deformations over time in the A549 spheroids. However, in the case of the SK-MES-1 spheroids, the presence of NFs and CAFs show a decline in the radial cumulative displacements (Fig. 3 e). The decline in the outward directed deformations can be either attributed to the reduced forces generated by the spheroids over time or the involvement of outward directed deformations. We also observed an increase in the invasiveness of the spheroids in both NFs and CAFs (Fig. 3 f), potentially hinting towards the escape of single cells into the ECM, in concurrence with the decline in the cumulative radial displacements.

To investigate the relationship between collagen deformation and the invasion potential of the cancer spheroids, we examined a time-dependent profile of the radial displacement and the invasive capability of the metastatic and non-metastatic cell lines (Fig. 3 c-f). The 2D projected area of cancer spheroids and the associated invaded cells over time was calculated to provide a measure of spheroid invasiveness. There is a clear negative correlation between the cell invasion area and collagen gel deformation over time in all cases. In the presence of stromal cells, we observed a lower collagen deformation and a larger spheroid invasion area, compared to the absence of stromal cells in the control. Stromal cells appear to modulate the rate of cell dissemination by enhancing the invasiveness of cancer cells through stromal cell-generated signals. In addition, we also observed a transition in the invasion pattern of cancer spheroids from collective to single cell invasion in the presence of stromal cells (Supplementary video 1, 2).

As expected, the metastatic cells (invasion index: 400.99 ± 42.16%) are more invasive than the non-metastatic cell lines (invasion index: 87.41 ± 7.60%), in agreement with the literature (Fig. 3 h,j, Supplementary Fig. 9 d,f). Also, the presence of a cellular TME in the form of stromal cells significantly increased the invasiveness of the cancer spheroids, in both the non-metastatic (ECs: 134.70 ± 6.76%, NFs: 121.57 ± 15.14%, CAFs: 160.82 ± 14.02%) and metastatic (ECs: 495.34 ± 11.20%, NFs: 519.09 ± 28.78%, CAFs: 577.89 ± 28.37%) cell lines after 24 h. In a similar fashion, the ECM deformations generated in the metastatic cells (Control: 5.75 ± 0.92, ECs: 6.54 ± 1.42, NFs: 1.47 ± 0.41, CAFs: 2.23 ± 0.31) were less in comparison to the non-metastatic (Control: 11.41 ± 0.82, ECs: 8.27 ± 1.30, NFs: 9.39 ± 0.79, CAFs: 5.02 ± 0.60) cell line (Fig. 3 g,i, Supplementary Fig. 9 c,e). We observed that CAFs induced maximum invasiveness and consistently low ECM deformations in both cancer cell types. It is likely that owing to the abundance and diverse functions of CAFs in the TME, they may produce factors that induce EMT (epithelial-to-mesenchymal transition) that enhance cell motility.

The following are the Supplementary data related to this article:

Video 1 Video 1

Video 2 Video 2

### 3.4 Collagen realignment and the identification of soluble factors affecting collagen deformation

**Fig. 4:**
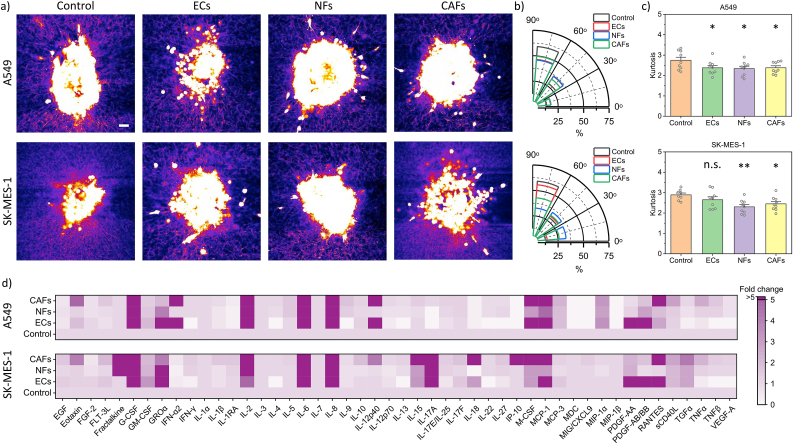
**Collagen realignment and cytokine assay.** (a) Confocal images showing collagen fibre alignment in the vicinity of the A549 (top row) and SK-MES-1 (bottom row) spheroids, comparing different stromal cell cases. Scale bar = 50 μm. (b) The relative frequency of the angular distribution of collagen fibrils is calculated within 100 μm of the spheroid surface in A549 (top) and SK-MES-1 (bottom) spheroids under various stromal cell conditions (for 0-30ᵒ, ECs (n.s.), NFs (***), CAFs (***); for 30ᵒ-60ᵒ, ECs (n.s.), NFs (*), CAFs (n.s.); for 60ᵒ-90ᵒ, ECs (n.s.), NFs (***), CAFs (***)). (c) Graph kurtosis in different stromal cell cases in A549 (top) and SK-MES-1 (bottom) spheroids. (d) Heatmap of a 48-Plex human cytokine array showing the relative fold change in cytokine/chemokine expression for different stromal cell cases in A549 (top) and SK-MES-1 (bottom) spheroids. Each data point represents average of spheroids per well. The plots and error bars represent mean ± s.e.m. for n = 9 samples and n.s. "not significant", *p < 0.05, **p < 0.01, and ***p < 0.001 (Student's t-test).

To validate the observed collagen displacements with the structural realignment of the collagen fibres, we stained the collagen fibres in the 24-well plate at the 24 h timepoint. We visualised the structural organisation of the collagen fibres near the metastatic vs non-metastatic cell spheroids (Fig. 4 a). The angular orientation of the fibres, within a 100 μm distance from the tumour boundary, was estimated. The simultaneous contraction of the ECM and tumour growth induced the reorientation of collagen at the spheroid surface. Fig. 4 b shows the relative frequency of the angular distribution of the collagen fibrils. For the A549 cells, from 0ᵒ-30ᵒ range (Control: 13.59 ± 0.01%, ECs: 17.62 ± 0.01%, NFs: 17.95 ± 0.01%, CAFs: 17.72 ± 0.01%) and from 60ᵒ-90ᵒ (Control: 59.10 ± 0.02%, ECs: 46.44 ± 0.02%, NFs: 45.80 ± 0.03%, CAFs: 49.52 ± 0.01%). For SK-MES-1, from 0ᵒ-30ᵒ (Control: 12.54 ± 0.01%, ECs: 14.25 ± 0.01%, NFs: 32.89 ± 0.03%, CAFs: 26.11 ± 0.01%) and 60ᵒ-90ᵒ (Control: 59.17 ± 0.02%, ECs: 55.63 ± 0.03%, NFs: 32.74 ± 0.03%, CAFs: 42.84 ± 0.02%). The majority of the collagen fibrils were aligned radially (between 60ᵒ and 90ᵒ) for all the stromal cell cases in both cancer cells, at the end of the experiment. Considering the entire dataset, the maximum alignment of collagen fibres was observed for both controls. Besides, the trend of the collagen displacement pattern (observed with fiducial beads) was largely consistent with the radial alignment of collagen fibrils in the control. The angular distribution was also evaluated in terms of their kurtosis coefficients (degree of peakedness of a distribution) as shown in Fig. 4 c and Supplementary Fig. 10. For non-metastatic cells (Control: 2.74 ± 0.15, ECs: 2.37 ± 0.11, NFs: 2.33 ± 0.11, CAFs: 2.38 ± 0.09) and for metastatic cells (Control: 2.81 ± 0.11, ECs: 2.65 ± 0.14, NFs: 2.30 ± 0.11, CAFs: 2.45 ± 0.11), the coefficients were calculated. The results were largely in agreement with the angular distribution plot in so far as the controls show maximum alignment in SK-MES-1 as well as A549 spheroids, when compared to the respective stromal cell cases. However, no profound difference was observed amongst the stromal cell cases.

Next, to elucidate the role of stromal cells in the apparent increase of cancer spheroid invasiveness, a multiplex cytokine assay was conducted. The growth cell culture media was collected after 24 h of the experiment and sent for the evaluation of a defined list of growth factors, cytokines, and chemokines. Fig. 4 d compares the fold change of the various cytokines/chemokines with the controls (Supplementary Fig. 11). We observe certain similarities in the overproduction of pro-inflammatory cytokines (such as IL-2, IL-6, IL-8, MCP-1, G-CSF) in the presence of stromal cells in both metastatic and non-metastatic spheroids. This may explain the pro-tumorigenic effect of stromal cells on cancer cell migration and invasiveness, as well as observed differences in the deformation field. We further supplemented the media in the control case with IL-6 (10 ng/ml, Bio-Techne) to see if it elicits similar response to CAFs case. We observed a significant increase in the invasion area in both metastatic and non-metastatic cell lines, however, the corresponding radial displacements although lesser than the control were not significantly different in both cell lines (Supplementary Fig. 12). This maybe a result of the compounding effect of a number of soluble factors that results in the observation of the negative correlation between collagen deformation and spheroid invasion ability. Thus, the upregulation of a number of cytokines, as a result of the interplay between stromal cells and cancer spheroids has been identified. Future investigation to underpin the secreting source of these key soluble factors would be interesting for drug discovery implications.

## 4 Discussion and conclusion

This work sought to capture the complexity of the TME by integrating a cellular microenvironment compartment of stromal cells within the current *in vitro* spheroid contractility assays. A layer of stromal cells (endothelial cells, normal fibroblasts, and cancer-associated fibroblasts) embedded in hydrogel was introduced onto a collagen gel comprising cancer spheroids and fluorescent beads, to track the deformations generated by the spheroids over a period of 24 h. The assay was used to elucidate the effect of stromal cells on the ability of tumour spheroids to deform collagen in both cases of metastatic and non-metastatic cancer cell lines. We observed that stromal cells increased the invasiveness of the cancer cells, while negatively influencing the physical pull exerted by the spheroids and the realignment of the collagen fibres due to a shift from collective to single-cell migration. In addition, we observed a concomitant upregulation of proinflammatory cytokines associated with cancer cell motility in conditions containing stromal cells, which correlated with the increased spheroid invasiveness observed in those particular experimental conditions, consistent with the literature [13]. Thus, cancer spheroid contractility and invasion is affected by the stromal cell populations in the TME, which modify the cancer cell behaviour through the secretion of pro-tumourigenic factors.

The CAFs case shows the overproduction of Eotaxin (CCL11) [28] and RANTES (CCL5) [29] in both metastatic and non-metastatic cell lines. These cytokines have been linked with promoting tumour aggressiveness (through increased motility and invasion) which is consistent with our observations. Several proinflammatory cytokines (IL-6, IL-8) secreted by the TME have been shown to accelerate tumour development and migration [13,30]. The transition of cancer cells from one state of migration to another has been shown to be governed by the mechanical (collagen density) and molecular (TGFβ) mechanisms in *in vitro* studies [31,32]. Our results seem to show a transition from collective to single cancer cell migration with the introduction of stromal cells. This observation may explain the negative correlation between cancer cell invasiveness and the deformation ability of cancer spheroids. While the collective migration of a cell cohort requires a significant remodelling of the ECM, the force generated in single cell migration is three orders of magnitude smaller than in collective tumour force generation [6,33,34]. Thus, we identified that the presence of proinflammatory cytokines may induce cancer cells to switch from a collective to a single cell invasion pattern.

Studies on traction forces have identified a positive correlation between the metastatic potential and cellular traction stresses in single cancer cells [3]. Here, we observed a negative correlation, potentially because of the difference in the scale (i.e., single cell vs multicellular) of the experiments. Our assay comprises multicellular tumour spheroids embedded in a hydrogel, thus providing a better representation of human solid tumours *in vivo* [35], to measure collective matrix deformations. We also employed the assay to compare the different behaviours of metastatic (SK-MES-1) and non-metastatic (A549) lung cancer cell lines. Metastatic cells are more invasive and migrated longer distances compared to non-metastatic primary tumours [36]. Our results are consistent with this observation, in so far as the invasion index in SK-MES-1 is higher than in A549 cell spheroids. In contrast, the deformations generated by SK-MES-1 cell spheroids are smaller than by A549 spheroids. These findings suggest that tumour spheroids generate inherently different collagen deformations depending on their metastatic potential. These differences could be used as a potential biophysical marker to diagnose the likelihood of metastasis.

Despite our multi-layer assay being an improvement over the current spheroid contractility assays, it notably only considers the non-direct stromal cell-tumour cell interactions through paracrine signalling unlike the *in vivo* situation which includes both paracrine as well as cell spatial arrangement. Thus, further future investigation by using co-culture of pre-mixed cancer and stromal cell spheroids has the potential to improve tumour pathophysiology. However, our current assay offers the possibility of parametric studies to understand, in detail, the soluble cross-talk between the cell types. The presence of stromal and tumour cells in direct contact will provide a significant advantage to emulate the TME and may alter the modes of migration of cancer cells. This, however, is outside the scope for the present study. Our assay was sensitive enough to capture the difference between CAFs and NFs. There was a pronounced change in collagen deformation in the case of CAFs compared to NFs. Additionally, a study showed a more coordinated and enhanced breast cancer cell invasion pattern in aligned collagen fibres, as opposed to single cell invasion in a randomly organised collagen matrix [37]. Our results (Fig. 4) agree with this observation, where single cancer cell invasion was visible in the presence of stromal cells, while the controls show more collagen alignment. Since the stromal cells were not in physical contact with the cancer spheroids to alter the collagen matrix [38], we speculate that the upregulation of MCP-1 in the stromal cell cases suggests the induction of MMP production by cancer cells that enable the cancer cells to break down the collagen matrix to invade [39,40]. We identified a preliminary role of MMP-2 and MMP-9 activity in SK-MES-1 and A549 spheroids respectively, in all stromal cell cases (Supplementary Fig. 13). Further quantification of MMPs and their corresponding inhibitors will give important insights into understanding their role in identifying the relationship between cancer cell invasion and collagen deformation.

In the context of spheroid contractility assays, we report a new, simple, and robust multilayer *in vitro* assay integrating multicellular cancer spheroids with the cellular TME to emulate the pathophysiology of cancer cell invasion behaviour. We observe a negative correlation between the cancer invasion index and collagen deformation and realignment. Furthermore, the presence of stromal cells switches the migratory behaviour of cancer cells from collective to single cell invasion, which is correlated to the upregulation of pro-inflammatory cytokines. Thus, stromal cells promote the detachment of cancer cells and alter the cancer cell's ability to manipulate the ECM. As cell-cell and cell-ECM interactions and matrix remodelling are gaining importance for therapeutic targeting, this study provides a simple assay to quantify the extent to which chemical signals (through tumour-stromal cell interactions) modulate the tumour-ECM mechanics.

## CRediT authorship contribution statement

**Ayushi Agrawal:** Methodology, Software, Validation, Formal analysis, Investigation, Resources, Data curation, Writing – original draft, Writing – review & editing, Visualization. **Soufian Lasli:** Methodology, Resources, Writing – review & editing. **Yousef Javanmardi:** Methodology, Software, Validation, Formal analysis, Investigation, Resources, Writing – review & editing, Visualization. **Diane Coursier:** Methodology, Resources, Data curation. **Auxtine Micalet:** Methodology, Software, Validation, Investigation, Resources, Data curation, Writing – original draft, Writing – review & editing, Visualization. **Sara Watson:** Resources, Data curation. **Somayeh Shahreza:** Resources, Data curation. **Bianca Serwinski:** Methodology, Software, Formal analysis, Resources, Data curation. **Boris Djordjevic:** Methodology, Software, Resources, Data curation. **Nicolas Szita:** Resources, Data curation. **Umber Cheema:** Supervision, Resources. **Sergio Bertazzo:** Methodology, Software, Investigation, Resources. **Fernando Calvo:** Supervision, Project administration, Writing – review & editing. **Emad Moeendarbary:** Conceptualization, Supervision, Project administration, Writing – review & editing.

## Declaration of competing interest

The authors declare that they have no known competing financial interests or personal relationships that could have appeared to influence the work reported in this paper.

## Data Availability

Data will be made available on request.

## References

[1] Agrawal A., Shahreza S., Javanmardi Y., Szita N., Moeendarbary E. (2022). The tumour microenvironment modulates cancer cell intravasation. Organs-on-a-Chip.

[2] Nia H.T., Munn L.L., Jain R.K. (2020). Physical traits of cancer. Science.

[3] Kraning-Rush C.M., Califano J.P., Reinhart-King C.A. (2012). Cellular traction stresses increase with increasing metastatic potential. PLoS One.

[4] Colin-York H. (2019). Spatiotemporally super-resolved volumetric traction force microscopy. Nano Lett..

[5] Li D. (2021). Astigmatic traction force microscopy (aTFM). Nat. Commun..

[6] Mark C. (2020). Collective forces of tumor spheroids in three-dimensional biopolymer networks. Elife.

[7] Chen Y.Q. (2019). Early stage mechanical remodeling of collagen surrounding head and neck squamous cell carcinoma spheroids correlates strongly with their invasion capability. Acta Biomater..

[8] Kopanska K.S., Alcheikh Y., Staneva R., Vignjevic D., Betz T. (2016). Tensile forces originating from cancer spheroids facilitate tumor invasion. PLoS One.

[9] Turley S.J., Cremasco V., Astarita J.L. (2015). Immunological hallmarks of stromal cells in the tumour microenvironment. Nat. Rev. Immunol..

[10] Watson S.A. (2022). Integrated role of human thymic stromal cells in hematopoietic stem cell extravasation. Bioeng. Transl. Med..

[11] Whelan I.T. (2023). A microphysiological model of bone development and regeneration. Biofabrication.

[12] Emon B., Bauer J., Jain Y., Jung B., Saif T. (2018). Biophysics of tumor microenvironment and cancer metastasis - a mini review. Comput. Struct. Biotechnol. J..

[13] Kartikasari A.E.R., Huertas C.S., Mitchell A., Plebanski M. (2021). Tumor-induced inflammatory cytokines and the emerging diagnostic devices for cancer detection and prognosis. Front. Oncol..

[14] Liubomirski Y. (2019). Tumor-stroma-inflammation networks promote pro-metastatic chemokines and aggressiveness characteristics in triple-negative breast cancer. Front. Immunol..

[15] Labernadie A. (2017). A mechanically active heterotypic E-cadherin/N-cadherin adhesion enables fibroblasts to drive cancer cell invasion. Nat. Cell Biol..

[16] Ansardamavandi A., Tafazzoli-Shadpour M. (2021). The functional cross talk between cancer cells and cancer associated fibroblasts from a cancer mechanics perspective. Biochim. Biophys. Acta, Mol. Cell Res..

[17] Sahai E. (2020). A framework for advancing our understanding of cancer-associated fibroblasts. Nat. Rev. Cancer.

[18] Naber H.P.H., Wiercinska E., ten Dijke P., van Laar T. (2011). Spheroid assay to measure TGF-β-induced invasion. J. Vis. Exp..

[19] Lang, N. R. et al. Estimating the 3D Pore Size Distribution of Biopolymer Networks from Directionally Biased Data. doi:10.1016/j.bpj.2013.09.038. 10.1016/j.bpj.2013.09.038 PMC3824714 24209841

[20] Liu Y. (2020). Fibrillar collagen quantification with curvelet transform based computational methods. Front. Bioeng. Biotechnol..

[21] Liu Y., Keikhosravi A., Mehta G.S., Drifka C.R., Eliceiri K.W. (2017). Methods for quantifying fibrillar collagen alignment. Methods Mol. Biol..

[22] Law J.X., Musa F., Ruszymah B.H.I., El Haj A.J., Yang Y. (2016). A comparative study of skin cell activities in collagen and fibrin constructs. Med. Eng. Phys..

[23] Hall C.M. (2023). Hippocampus of the APPNL–G–F mouse model of Alzheimer's disease exhibits region-specific tissue softening concomitant with elevated astrogliosis. Front. Aging Neurosci..

[24] Whisler J. (2023). Emergent mechanical control of vascular morphogenesis. Sci. Adv..

[25] Javanmardi Y. (2023). Endothelium and subendothelial matrix mechanics modulate cancer cell transendothelial migration. Adv. Sci..

[26] Malandrino A., Mak M., Kamm R.D., Moeendarbary E. (2018). Complex mechanics of the heterogeneous extracellular matrix in cancer. Extrem. Mech. Lett..

[27] Javanmardi Y., Colin-York H., Szita N., Fritzsche M., Moeendarbary E. (2021). Quantifying cell-generated forces: Poisson's ratio matters. Commun. Phys..

[28] Huang W.Y. (2022). Cancer-associated fibroblasts promote tumor aggressiveness in head and neck cancer through chemokine ligand 11 and C-C motif chemokine receptor 3 signaling circuit. Cancers.

[29] Xu H. (2022). Cancer associated fibroblast–derived CCL5 promotes hepatocellular carcinoma metastasis through activating HIF1α/ZEB1 axis. Cell Death Dis..

[30] Greten F.R., Grivennikov S.I. (2019). Inflammation and cancer: triggers, mechanisms, and consequences. Immunity.

[31] Mehta P., Rahman Z., Dijke P., Boukany P.E. (2022).

[32] Wu J. (2021). Plasticity of cancer cell invasion : patterns and mechanisms. Transl. Oncol..

[33] Lintz M. (2017).

[34] Trepat X. (2009). Physical forces during collective cell migration. Nat. Phys..

[35] Nunes A.S., Barros A.S., Costa E.C., Moreira A.F., Correia I.J. (2019). 3D tumor spheroids as in vitro models to mimic in vivo human solid tumors resistance to therapeutic drugs. Biotechnol. Bioeng..

[36] Welch D.R., Hurst D.R. (2019). Defining the hallmarks of metastasis. Cancer Res..

[37] Riching K.M. (2014). 3D collagen alignment limits protrusions to enhance breast cancer cell persistence. Biophys. J..

[38] Glentis A. (2017). Cancer-associated fibroblasts induce metalloprotease-independent cancer cell invasion of the basement membrane. Nat. Commun..

[39] Wang L., Lan J., Tang J., Luo N. (2022). MCP-1 targeting: shutting off an engine for tumor development. Oncol. Lett..

[40] Friedl P., Wolf K. (2008).

