# Supplementary material for: Stromal cells regulate mechanics of tumour spheroid
Source: Mater Today Bio. 2023 Oct 6;23:100821. doi: 10.1016/j.mtbio.2023.100821 (PMC10585335; doi:10.1016/j.mtbio.2023.100821)
Supplement: Multimedia component 1 [file mmc1.docx]

**Supplementary information: Stromal cells regulate mechanics of tumour spheroid**

Ayushi Agrawal^1^, Soufian Lasli^1^, Yousef Javanmardi^1^, Diane Coursier^4^, Auxtine Micalet^5^, Sara Watson^1^, Somayeh Shahreza^1^, Bianca Serwinski^1,6,7^, Boris Djordjevic^1,6^, Nicolas Szita^2^, Umber Cheema^5^, Sergio Bertazzo^8^, Fernando Calvo^4^, Emad Moeendarbary^1,3*^

^1^Department of Mechanical Engineering, University College London, London WC1E 7JE, UK

^2^Department of Biochemical Engineering, University College London, London WC1E 7JE, UK

^3^Department of Biological Engineering, Massachusetts Institute of Technology, Cambridge, Massachusetts 02139, USA

^4^Instituto de Biomedicina y Biotecnología de Cantabria (Consejo Superior de Investigaciones Científicas, Universidad de Cantabria), Santander, Spain

^5^Department of Targeted Intervention, Division of Surgery and Interventional Science, University College London, London WC1E 7JE, UK

^6^199 Biotechnologies Ltd, Gloucester Road, London, W2 6LD, UK

^7^Faculty of Social Sciences, Northeastern University London, London, E1W 1LP, UK.

^8^Department of Medical Physics and Biomedical Engineering, University College London, London WC1E 6BT, UK

^*^Correspondence: [e.moeendarbary@ucl.ac.uk](mailto:e.moeendarbary@ucl.ac.uk)

University College London
Department of Mechanical Engineering
Roberts Engineering Building
Torrington Place,
London WC1E 7JE, UK

+44 (0) 20 7679 4125

**Supplementary information**

**Materials and Methods**

**Immunostaining**

For staining of the stromal cells (ECs, NFs, and CAFs) embedded in fibrin gel, gels were fixed with 4% PFA at the required time point. Cells were permeabilised using 0.1% Triton-X and were subsequently washed. Following this, cells were stained with DAPI (Cell Signaling Technology) and Alexa Fluor 647-Phalloidin (Thermo Fisher) overnight. Gels were then washed with PBS and imaged.

**Imaging and analysis**

An upright confocal microscope was employed to image the stromal cells in the top layer of the multilayer assay. A 25x water objective was used to capture z-stack images, every 10 µm apart.

A maximum projection of the images was performed and cell number was measured by counting the number of DAPI positive cells in the field of view of an image.

**Sample preparation for SEM imaging**

Collagen (2 mg/ml) and fibrin (3 mg/ml) hydrogels were prepared in glass bottomed dishes (MatTek Corp, US) after treating the glass surface with 5% glutaraldehyde for 30 mins. The hydrogels were then fixed using 4% paraformaldehyde and washed thoroughly with de-ionised water. The samples were serially dehydrated by 30%, 50%, 70%, 90%, and 100% (x3) graded ethanol, each step for 20 min. After that, specimens were immersed in 100% hexamethyldisilane (HMDS) and air dried. Next, the glass portion of the dish was separated from the dish using tweezers by applying a gentle pressure on it. The glass part containing the samples were attached using carbon tape to a SEM stub and sputter coated with carbon and platinum. A Carl Zeiss LEO 1530 were used at accelerating voltages of 5kV for SEM imaging with secondary electron (SE) mode.

**Fibre diameter and pore size calculation from SEM images**

Fibre diameter was calculated using the straight-line tool in Fiji. At least 20 measurements were performed for each ROI (Region of Interest) and three images were analysed per dish.

To measure the pore area, the images were binarised using “Trainable Weka Segmentation” plugin in Fiji. The area of each pore was calculated using “Analyse Particle” tool in Fiji.

**Atomic Force Microscopy (AFM)**

AFM indentation tests to characterise the mechanical properties of the hydrogels has been described in detail, previously ^1,2^. Briefly, AFM force-distance plots were acquired using a JPK Nanowizard Cellhesion 200 (JPK Instrument, Germany). The nominal spring constant of the tipless cantilever was 0.07 Nm^-1^ and 25 µm diameter glass microspheres were glued to the tip for indentation. The elastic modulus of the acellular hydrogels was carried out on about 15 randomly selected points in each dish and the average of these values were reported as the sample’s elastic modulus as data points in Supplementary Figure 4. A total of 8 samples were used for each condition.

**Microfluidic device fabrication for permeability measurement**

The protocol to fabricate the devices to measure permeability of the acellular hydrogels has been mentioned in detail, previously ^1^. Briefly, acrylic moulds were designed (50 mm x 6 mm x 1 mm) using AutoCAD software and printed on 1 mm thick acrylic sheets using a laser cutter (Epilog). The cut-out moulds were glued to a petri dish to further fabricate PDMS (Polydimethylsiloxane, Sylgard-184, Dow Corning) devices. The PDMS devices (PDMS:Curing agent = 10:1) were mixed, degassed, and poured over the moulds upto a height of 4mm. After curing the devices for 1.5 hr at 80° C, the PDMS was cut and peeled from the mould. Holes of 2mm and 4mm were punched on either end of the gel channel using biopsy punches (Miltex). The devices were then cleaned and bonded with a glass coverslip using a plasma gun for 1 min. The devices were further kept in the oven (80° C) overnight to restore the hydrophobicity of the PDMS and glass surface and create an irreversible bonding. Next, the hydrogel solution was injected into the gel channel from the smaller port, followed by hydrogel incubation 37° C. Finally, to measure permeability, about 50 µl dye was added to the larger port and the devices were imaged every 15 min.

**Zymography**

The protocol for MMP2 and MMP9 activity quantification has been described previously ^3^. Briefly, cell culture media collected after 24 hour of the experiment from the well plates was concentrated using Amicon Ultra-2 Centrifugal Filter Units, of 30 kDa cutoff (Sigma–Aldrich, Dorset, UK). The concentrated media was diluted 1:1 with NovexTris-Glycine SDS sample buffer. A precast Novex 0.1% gelatin zymogram gel was loaded with 20 µl of sample and was run at 125V for 2hr. The gel was then incubated for 30 min in renaturing buffer, followed by 30 min incubation in developing buffer, and finally overnight in fresh developing buffer in a humid incubator. The next day, the gel was stained with SimplyBlue SafeStain after washing the gel x3 with DI water. The gel was then imaged using an Epson Perfection V39 scanner (Epson, Nagano, Japan). All reagents and precast gels were purchased from Invitrogen.

**Supplementary Figure**

**Supplementary Figure 1: Spheroid characterisation.** The graph in a) represents the A549 spheroid circularity and in b) spheroid volume at t=1hr post embedding in the collagen gel. Each data point represents the average of 3 spheroids per well. The plots and error bars represent mean ± s.e.m. for n = 10 samples.


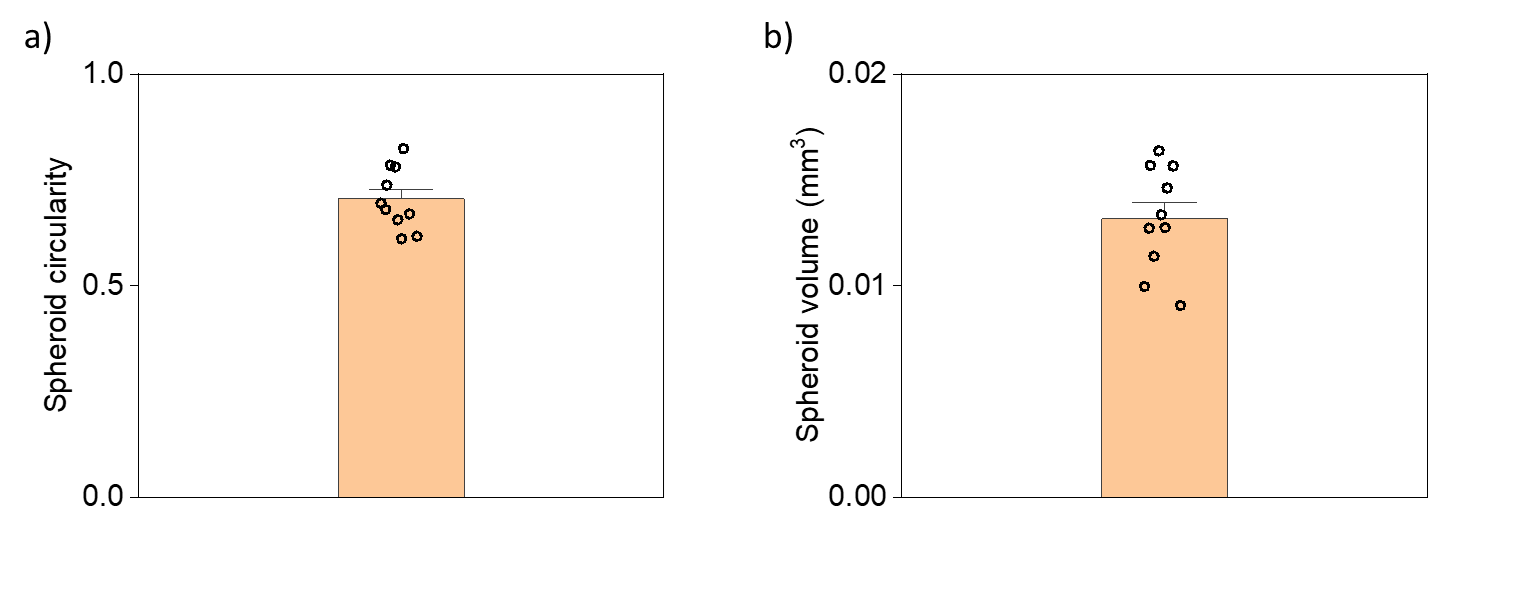


**Supplementary Figure 2: Stromal cell behaviour in fibrin gel.** a) Confocal images representing different stromal cells (ECs, NFs, CAFs) at t=6hr and 24hr after embedding in fibrin hydrogel. The cells were stained with DAPI (cyan) and F-actin (yellow). Scale bar = 50 µm. b) Graphs showing cell counts per image field of view of different stromal cells. The plots and error bars represent mean ± s.e.m. for n = 4 samples and ‘n.s.’ p > 0.05, *p < 0.05, **p<0.01, and ***p<0.001 (Student’s t-test).


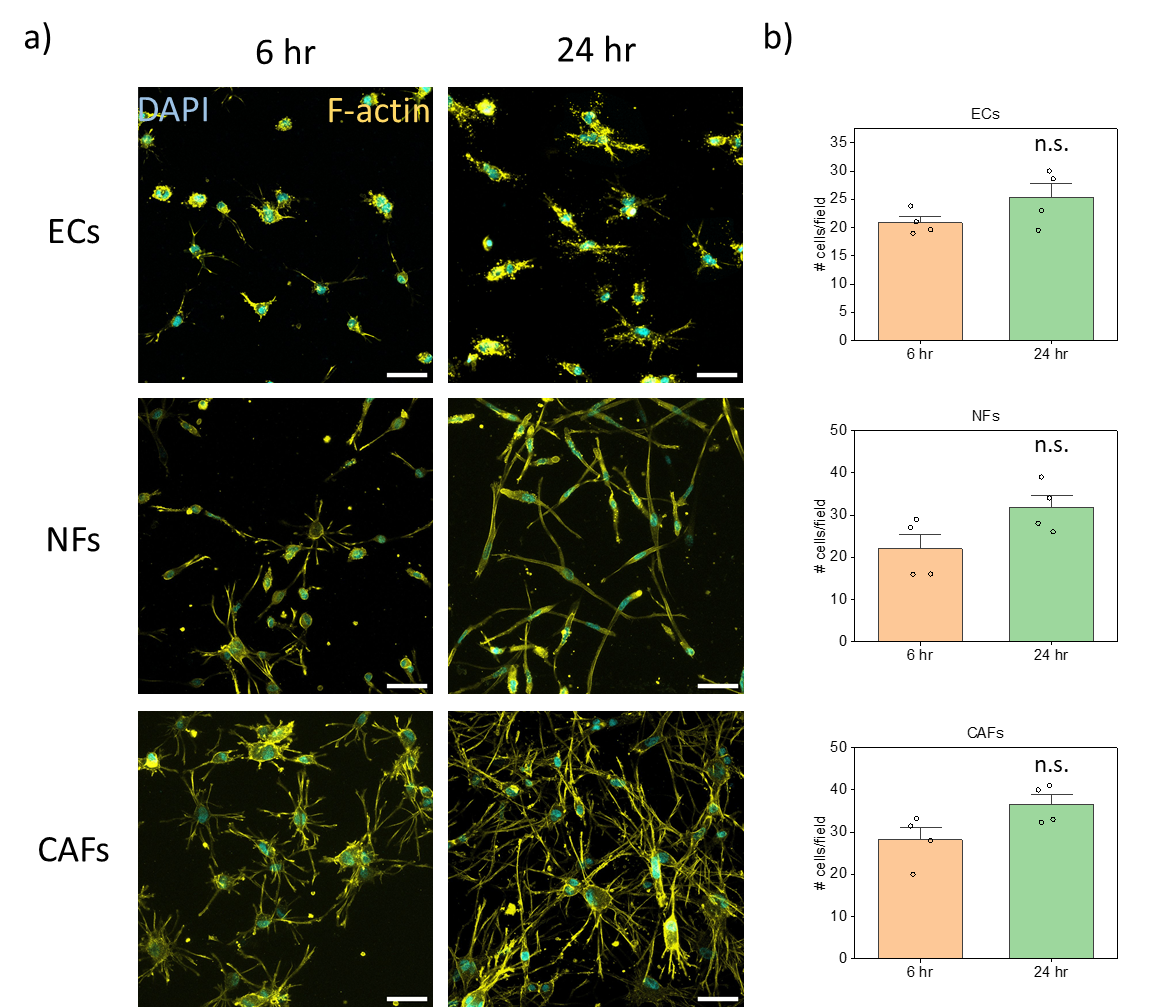


**Supplementary Figure 3: Scanning Electron Microscopy (SEM) images of the acellular collagen and fibrin hydrogels.** a) shows representative SEM images of acellular collagen and fibrin hydrogels. Scale bar - 200nm. The graph in b) shows comparison of fibre diameter (n=4) between collagen and fibrin fibres in nm. The graph in c) shows the pore area (n=4) of collagen and fibrin pores in µm^2^. Each data point represents an average of 3 images per dish. The plots and error bars represent mean ± s.e.m. and n.s. "not significant", *p < 0.05, **p<0.01, and ***p<0.001 (Student’s t-test).

**
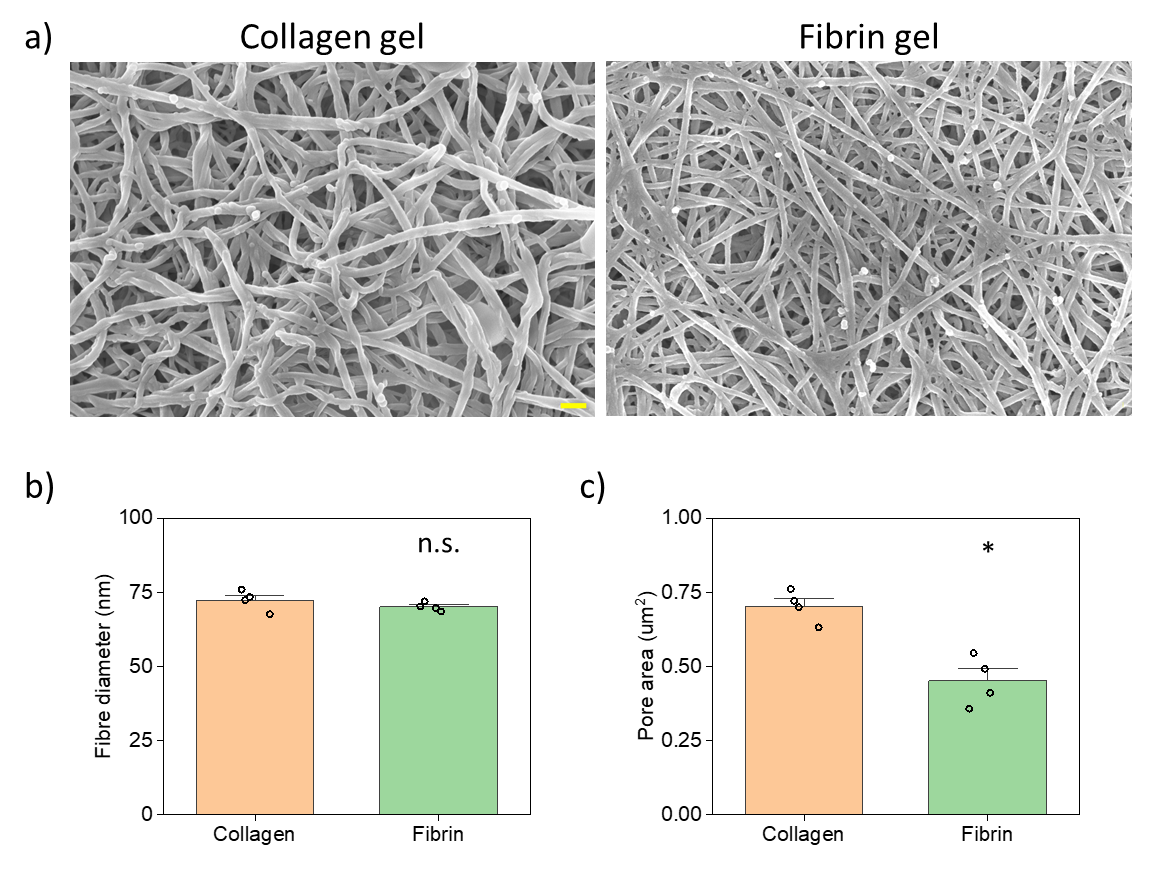
**

**Supplementary Figure 4: Stiffness measurement of acellular hydrogels.** a) Schematic representing the AFM (Atomic Force Microscopy) setup. b) Graph showing elastic modulus measurements of the acellular hydrogels using AFM indentation tests on the top surface of the gels (n=8). Each data point represents an average of 15 measurements per dish. The plot and error bars represent mean ± s.e.m. and n.s. "not significant", *p < 0.05, **p<0.01, and ***p<0.001 (Student’s t-test).

**
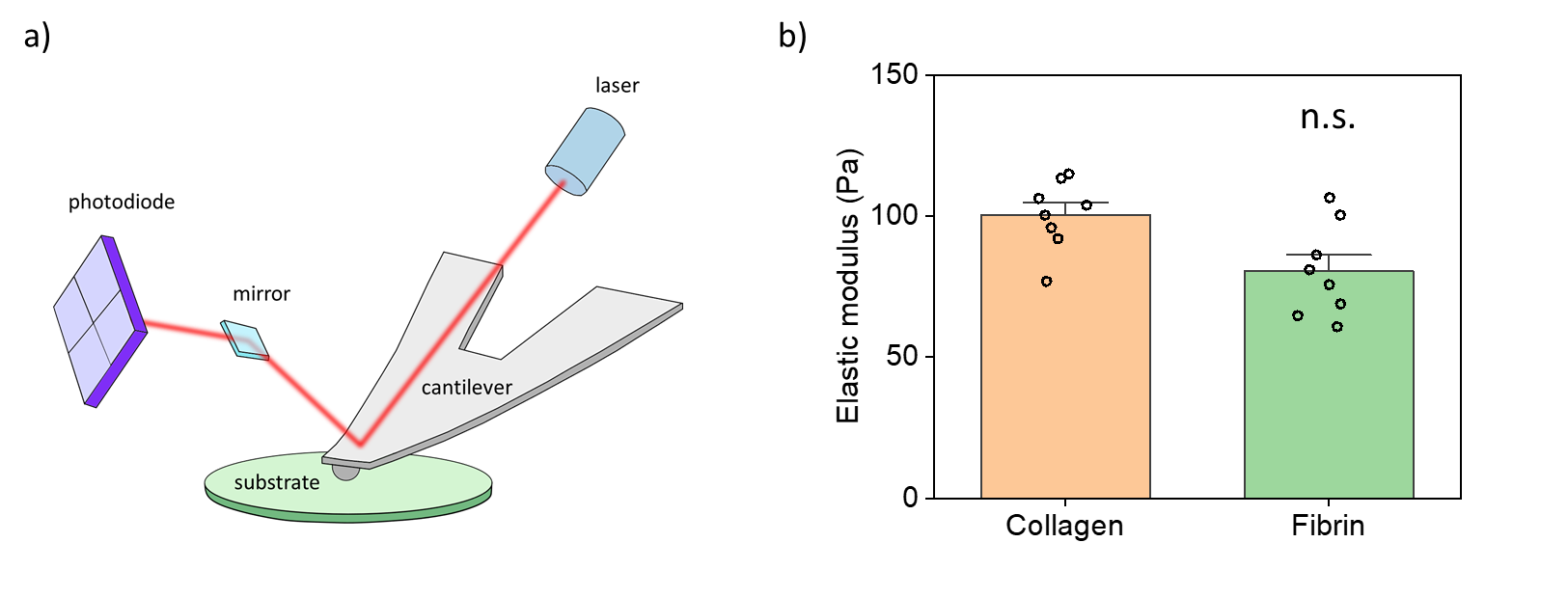
**

**Supplementary Figure 5: Permeability measurement for acellular hydrogels.** a) shows representative time-lapse images of the dye (in blue) penetration into the gel channel along the length of the channel. Scale bar 1cm. b) shows quantification of the distance travelled by the dye (x, cm) vs time. c) shows graph of the distance covered by the dye after 2 hr for collagen and fibrin hydrogels (n=3). Each data point is an independent experiment. The plot and error bars represent mean ± s.e.m. and n.s. "not significant", *p < 0.05, **p<0.01, and ***p<0.001 (Student’s t-test).

**
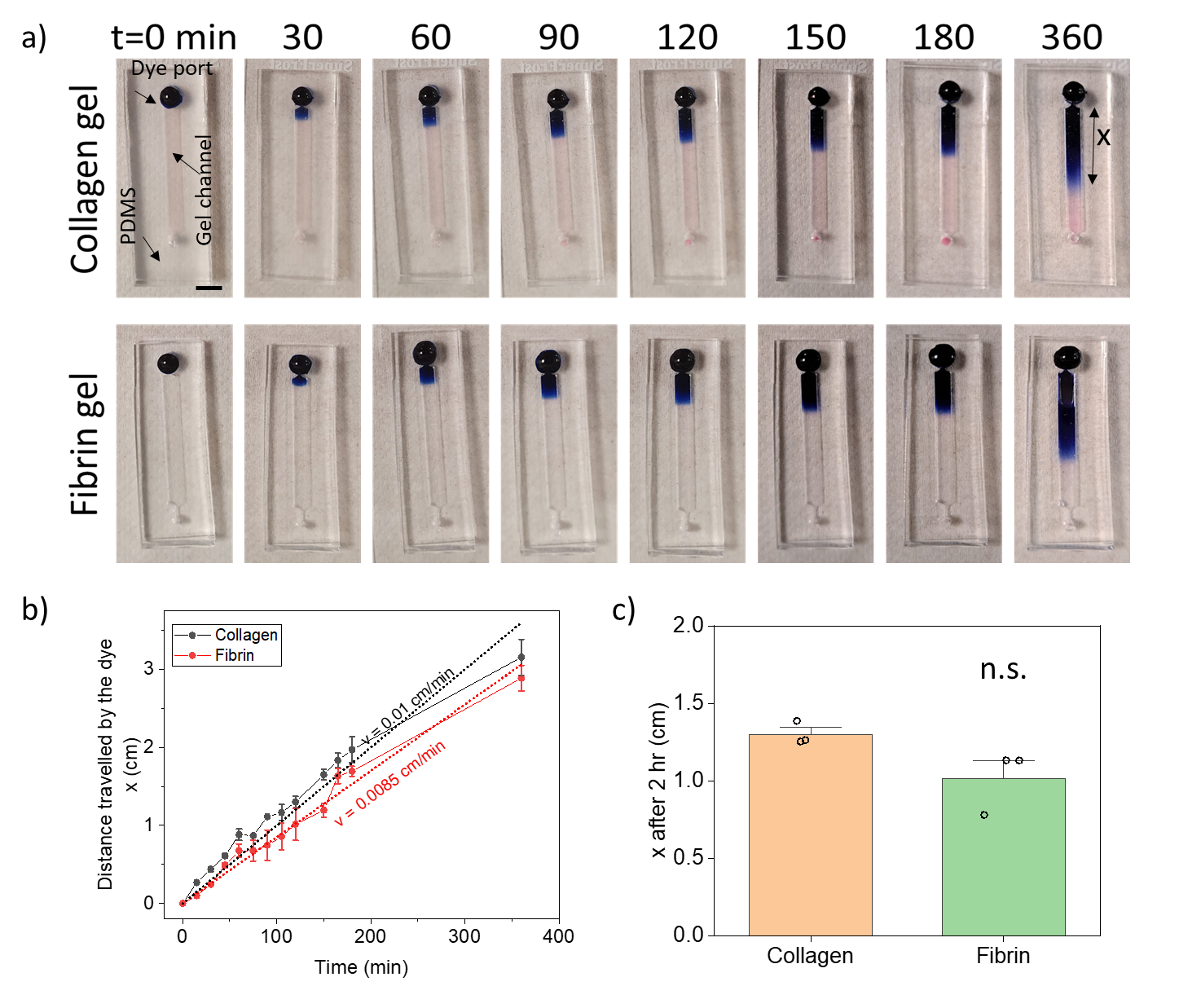
**

**Supplementary Figure 6:** a) shows an orthogonal projection view of the entire span of the multilayer gels using a confocal microscope (5x objective). The gels were stained with TAMRA dye. Scale bar = 500 µm. b) shows the graph of the gel layer heights (n=4). Each data point is an average of 5 height measurements at random locations per well. The plot and error bars represent mean ± s.e.m.


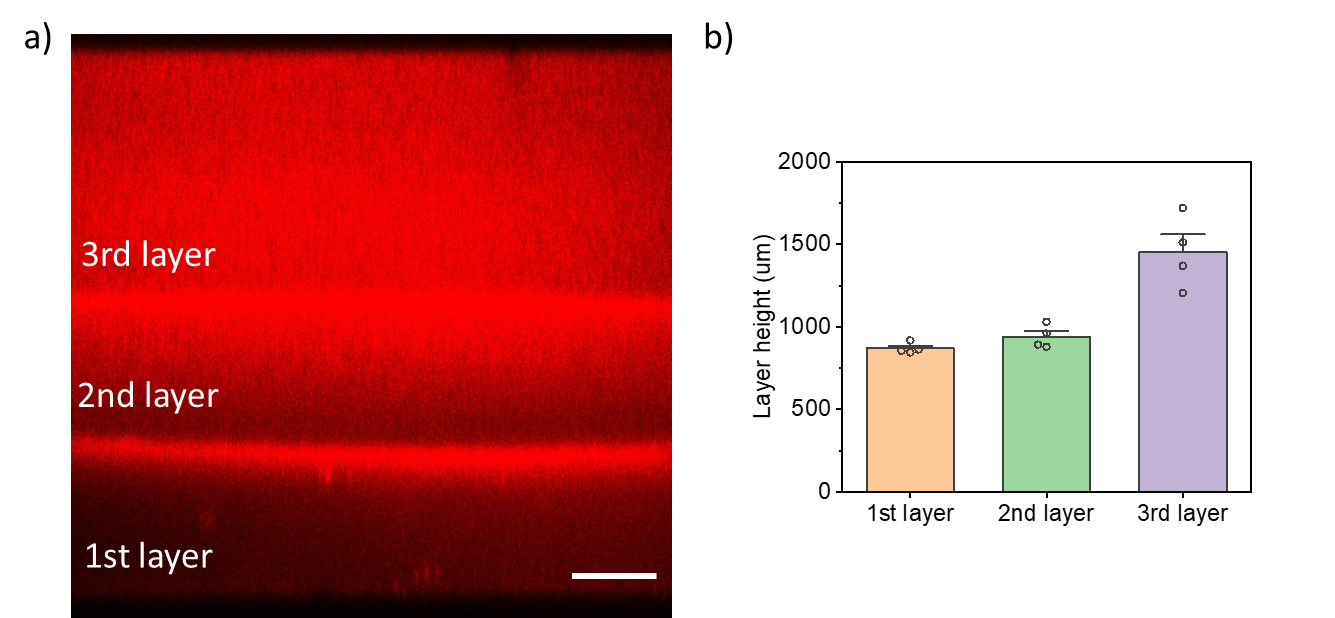


**Supplementary Figure 7: Raw displacements of A549.** Graphs representing the magnitude of radial raw displacements of collagen deformation by A549 cells in a) Control (n=14), b) ECs (n=8), c) NFs (n=14), d) CAFs (n=8). The plots and error bars represent mean ± s.e.m.


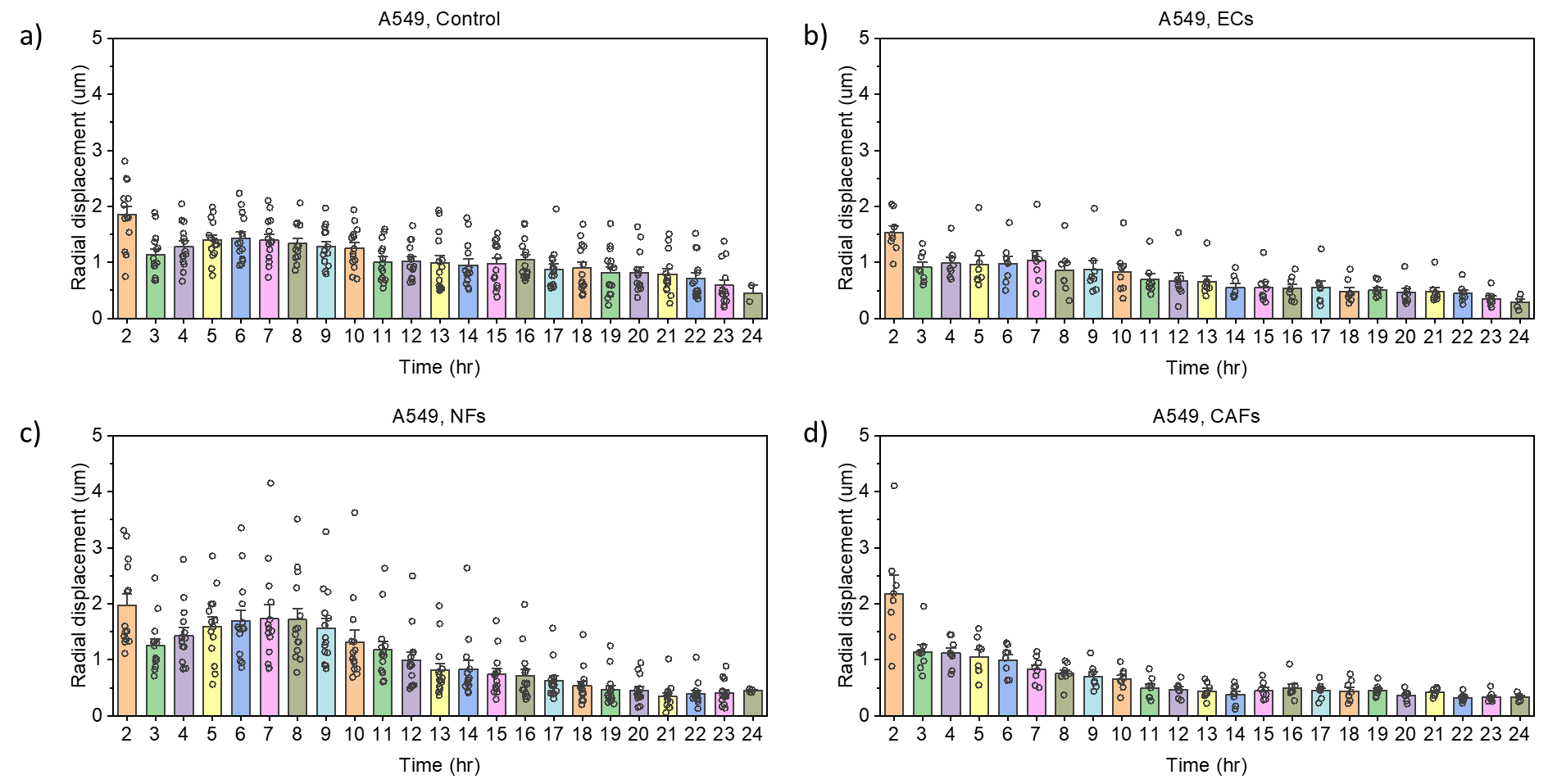


**Supplementary Figure 8: Raw displacements of SK-MES-1.** Graphs representing the magnitude of radial raw displacements of collagen deformation by SK-MES-1 cells in a) Control (n=7), b) ECs (n=7), c) NFs (n=9), d) CAFs (n=8). The plots and error bars represent mean ± s.e.m.


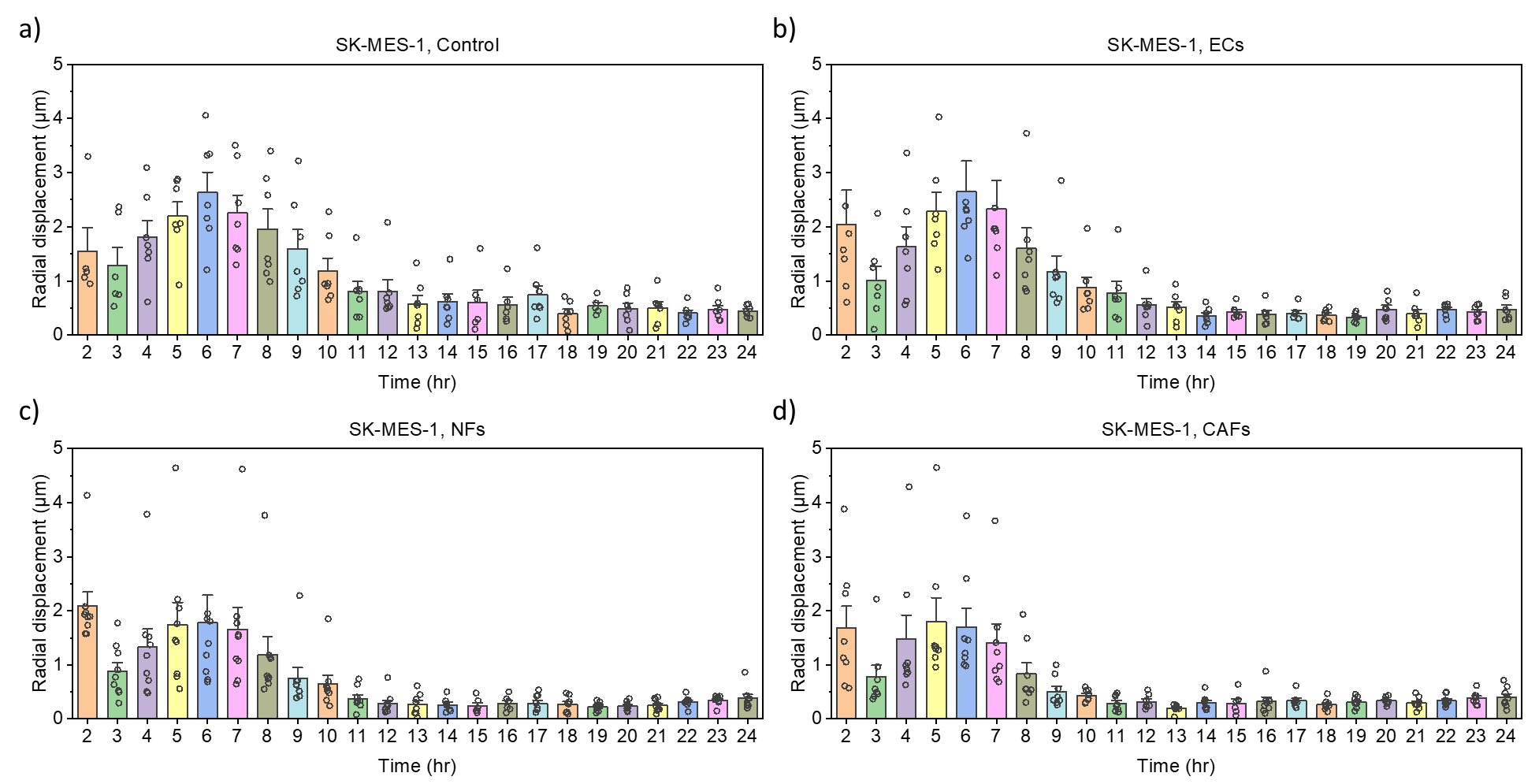


**Supplementary Figure 9: Quantitative plots comparing collagen deformation and invasion of metastatic (SK-MES-1) and non-metastatic (A549) spheroids.** (a&b) Change in normalised radial displacement between t=23 hr and t=2 hr over the normalised distance from the spheroid centre for different cases of stromal cells in (a) A549 spheroids and (b) SK-MES-1 spheroids. (c&e) Average normalised displacement at t=23 hr in (c) A549 spheroids and (e) SK-MES-1 spheroids. (d&f) Invasion area (%) at t=23 hr in (d) A549 spheroids and (f) SK-MES-1 spheroids. Each data point represents average of spheroids per well. The plots and error bars represent mean ± s.d. for A549 (n=14 for Ctrl, n=8 for ECs, n=12 for NFs, n=8 for CAFs) and SK-MES-1 (n=7 for Ctrl, n=7 for ECs, n=9 for NFs, n=8 for CAFs) spheroids and *p < 0.05, **p<0.01, and ***p<0.001 (Student’s t-test).


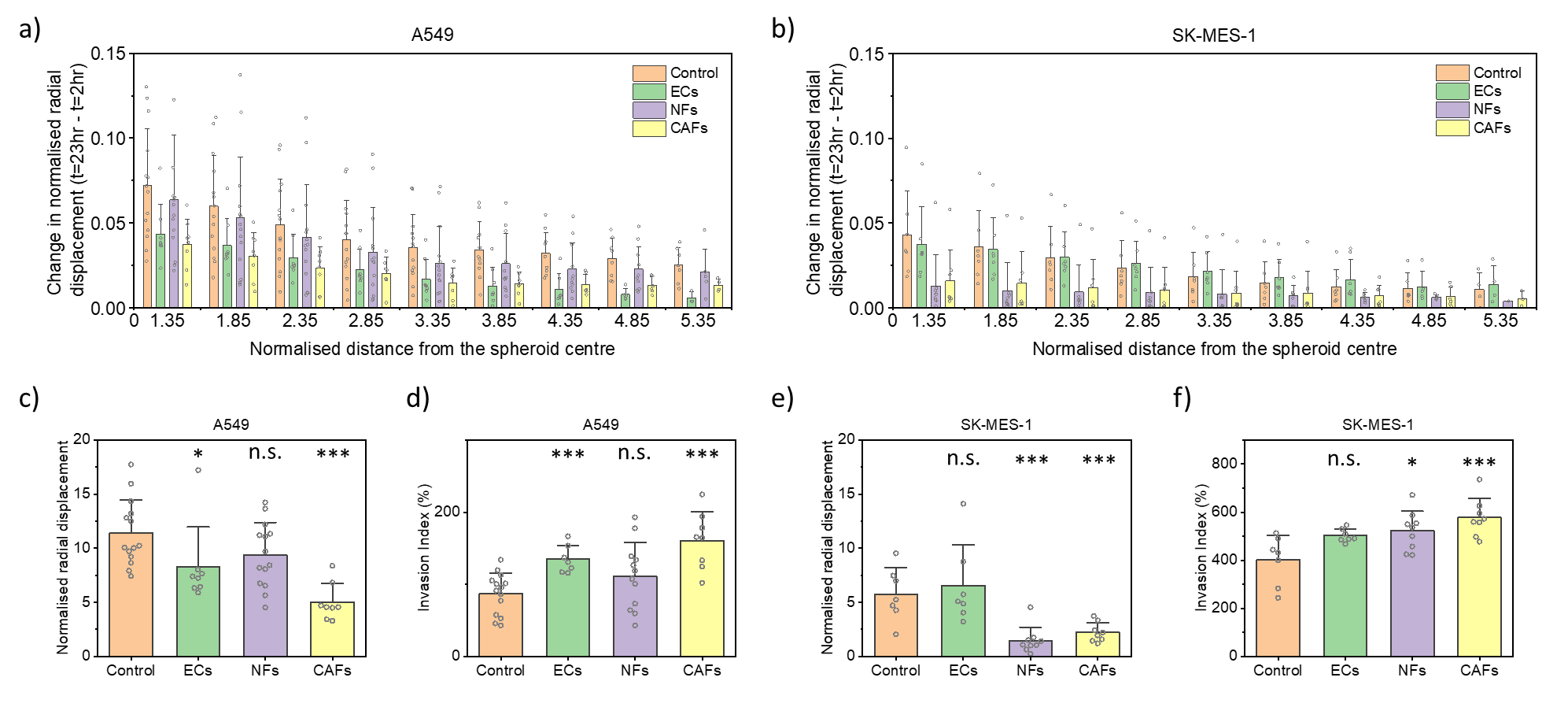


**Supplementary Figure 10: Collagen realignment.** Graph showing kurtosis in different stromal cell cases in (a) A549 and SK-MES-1 spheroids. Each data point represents average of spheroids per well. The plots and error bars represent mean ± s.d. for n = 9 samples and n.s. "not significant", *p < 0.05, **p<0.01, and ***p<0.001 (Student’s t-test).


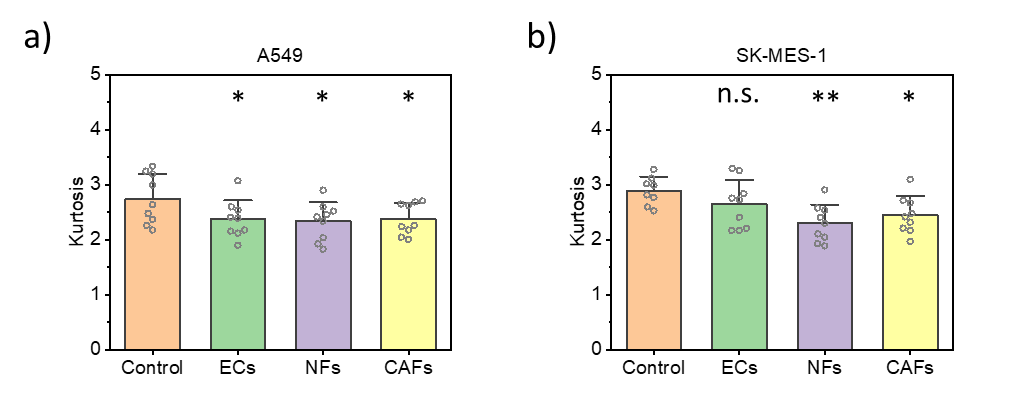


**Supplementary Figure 11: Multiplex assay for some soluble factors.** The figure shows fold change w.r.t control of a number of soluble factors in a) A549 and b) SK-MES-1 spheroids in the presence of different stromal cells.


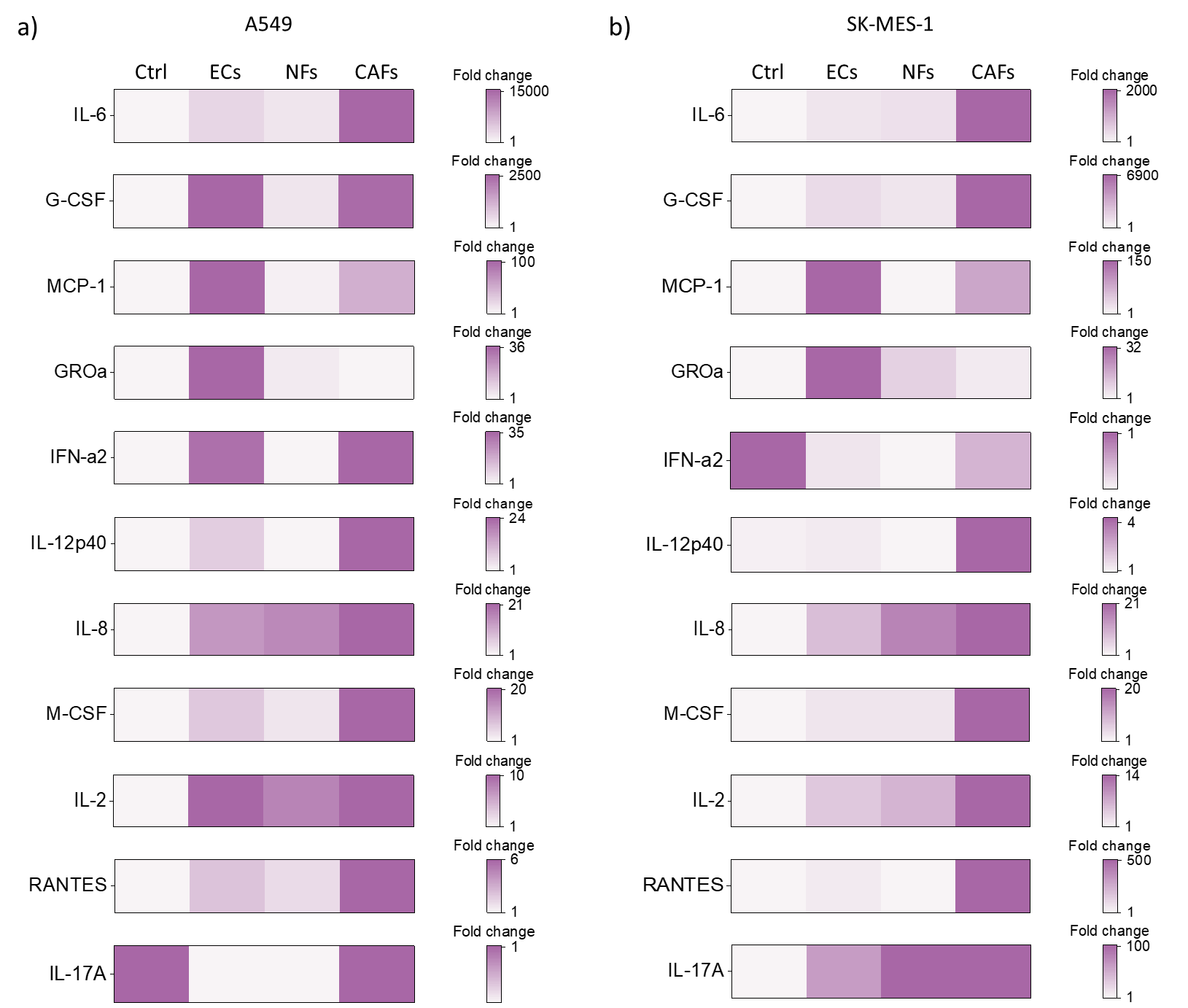


**Supplementary Figure 12: Effect of IL-6 dosing on collagen deformation and spheroid invasion.** Cumulative quantitative 2D plots of bead displacement at t=2 hr, 12 hr, and 23 hr comparing a) A549 spheroids and b) SK-MES-1 spheroids in control and IL-6 (10 ng/ml) supplemented media. Graphs showing progression of normalised radial displacement (left) and invasion area (right) with time in c) A549 spheroids and b) SK-MES-1 spheroid case. The plots and error bars represent mean ± SD. Bar plots of normalised radial displacement and invasion area at t=24 hr in e) A549 spheroids and f) SK-MES-1 spheroids. Each data point represents an average of 3 spheroids per well. The plots and error bars represent mean ± s.e.m. for n = 3 samples and n.s. "not significant", *p < 0.05, **p<0.01, and ***p<0.001 (Student’s t-test).

**
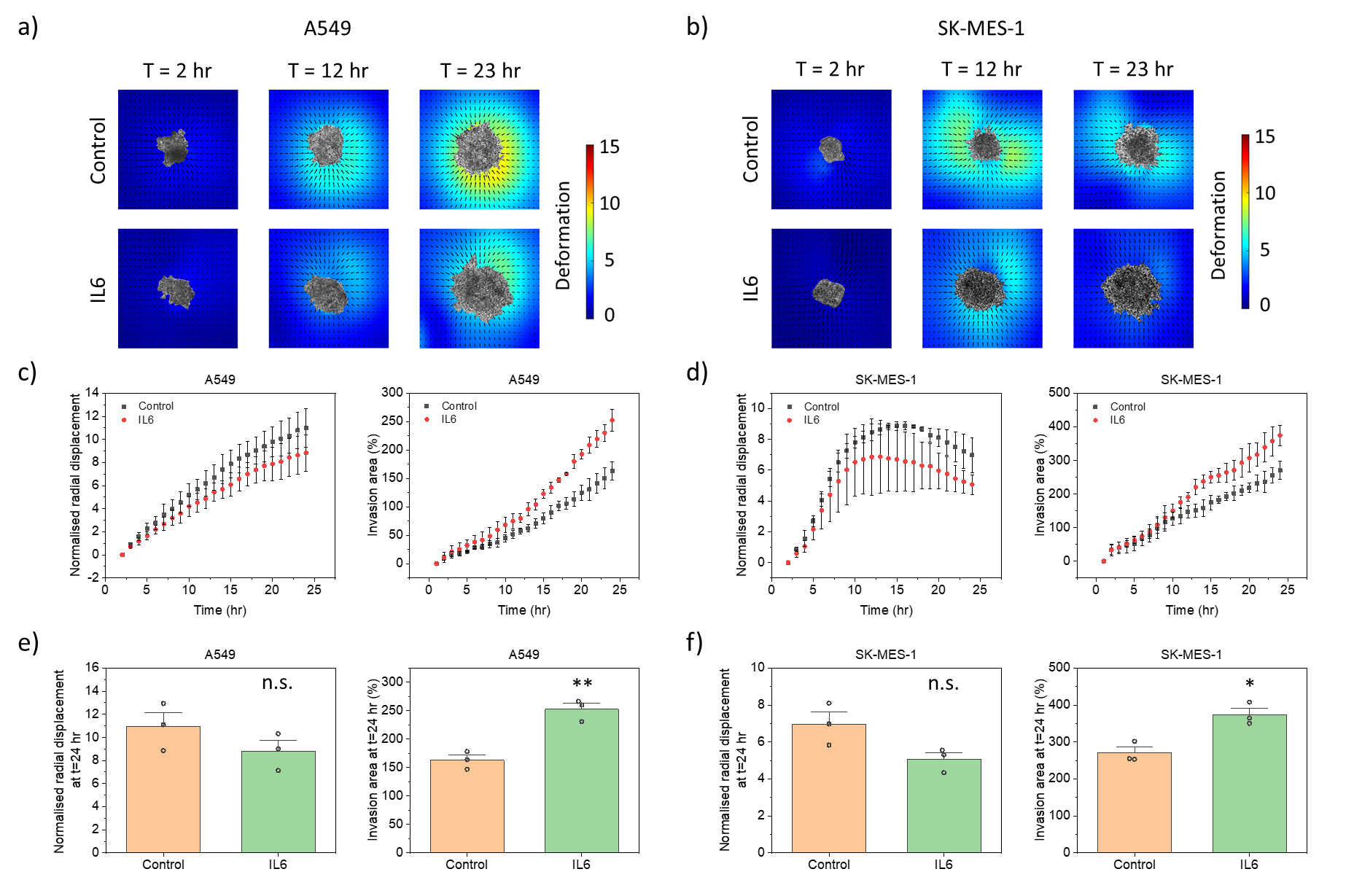
**

**Supplementary Figure 13: MMP2 and MMP9 activity in the presence of stromal cells in A549 and SK-MES-1 spheroids.** Zymography showing the presence and activity of MMP-9 and MMP-2 in different stromal cell conditions of a) A549, d) SK-MES-1 spheroids after 24 hrs. Quantification of fold change MMP2 band intensity in different stromal cell conditions of b) A549, e) SK-MES-1 spheroids. Plot showing fold change of MMP9 band intensity in different stromal cell conditions of c) A549, f) SK-MES-1 spheroids. The plots and error bars represent mean ± s.e.m and n.s. "not significant“, *p < 0.05, **p<0.01, and ***p<0.001 (Student’s t-test).

**
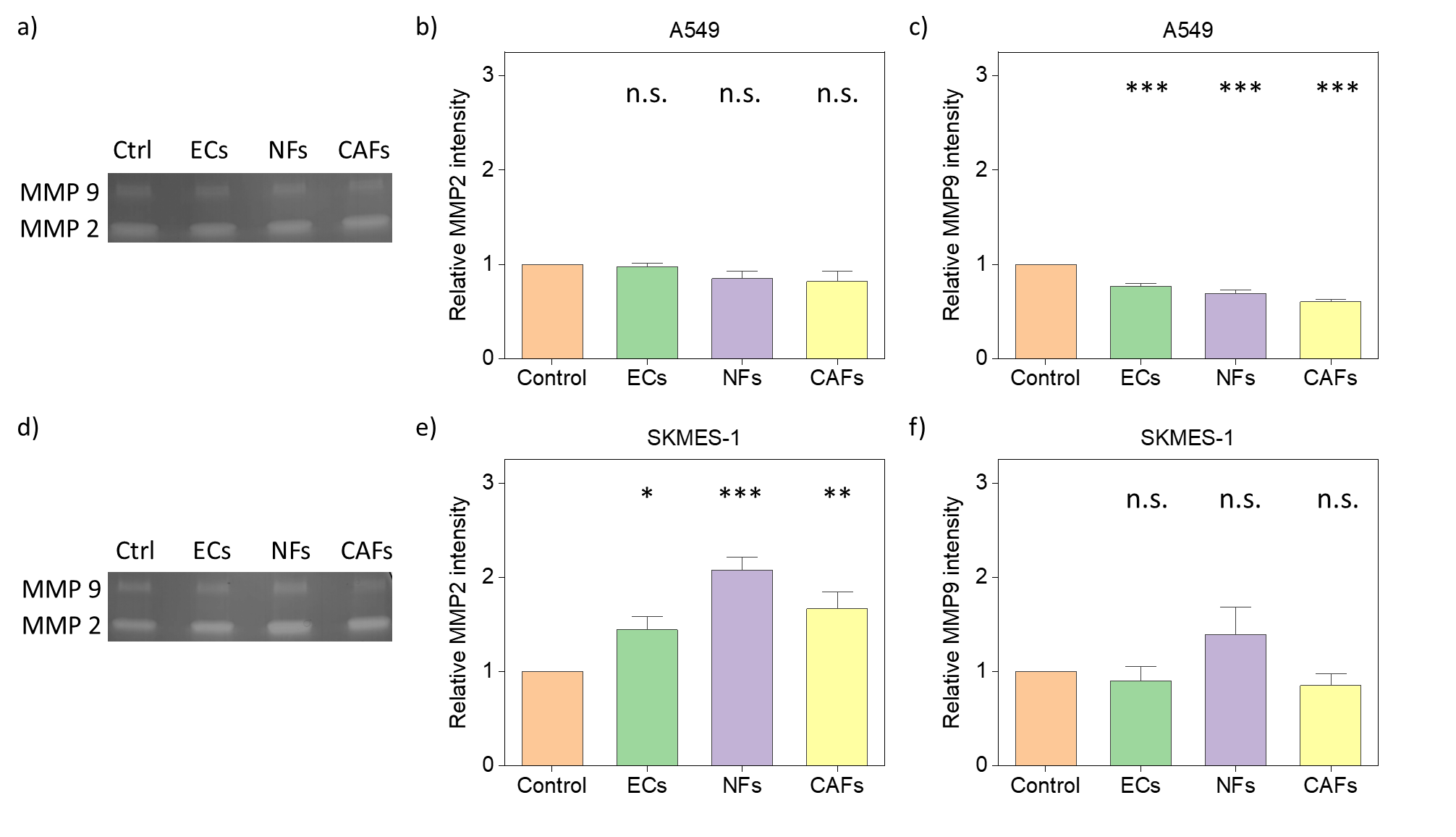
**

**References**

1. Javanmardi, Y. *et al.* Endothelium and Subendothelial Matrix Mechanics Modulate Cancer Cell Transendothelial Migration. *Adv. Sci.* **10**, 2206554 (2023).

2. Hall, C. M. *et al.* Hippocampus of the APPNL–G–F mouse model of Alzheimer’s disease exhibits region-specific tissue softening concomitant with elevated astrogliosis. *Front. Aging Neurosci.* **15**, (2023).

3. Micalet, A. *et al.* Evaluating the Impact of a Biomimetic Mechanical Environment on Cancer Invasion and Matrix Remodeling. *Adv. Healthc. Mater.* 2201749 (2022). doi:10.1002/ADHM.202201749
